# Multi-Omics Dissection and Functional Validation of Candidate Regulators Modulating Stress Tolerance and Xylose Utilization in the Natural Yeast Strain YB-2625

**DOI:** 10.3390/jof12090631

**Published:** 2026-08-23

**Authors:** Cheng Cheng, Teng-Fei Wu, Hong-Lei Mao, Wei-Bin Wang, Xin-Qing Zhao

**Affiliations:** 1 School of Biotechnology and Food Engineering, Hefei Normal University, Hefei 230601, China; chengcheng1990119@hfnu.edu.cn (C.C.); mhl07170526@outlook.com (H.-L.M.); 2 State Key Laboratory of Microbial Metabolism, School of Life Sciences and Biotechnology, Shanghai Jiao Tong University, Shanghai 200240, China; tfwu82@163.com (T.-F.W.); wangweibin@sjtu.edu.cn (W.-B.W.)

**Keywords:** *Saccharomyces cerevisiae*, xylose metabolism, natural isolate, multi-omics, epigenetic regulation

## Abstract

The intrinsic weakness of the budding yeast *Saccharomyces cerevisiae* in xylose utilization limits its application in biological manufacturing using lignocellulosic biomass. Although the natural yeast strain *S. cerevisiae* YB-2625 exhibits superior innate xylose-fermenting capability, the underlying mechanisms remain largely unexplored. Here, we employed comparative multi-omics to systematically dissect the molecular basis of its high stress tolerance and superior xylose consumption. Comparative genomics revealed 73,842 single nucleotide polymorphisms (SNPs) and 5191 small insertions/deletions (InDels) in YB-2625 relative to S288C, with significant enrichment in genes associated with chromatin remodeling, transcriptional regulation, and stress signaling. Integration of genomic and transcriptomic data identified candidate variants in key regulators. Functional validation further demonstrated that Tra1, a component of the SAGA, SLIK, and NuA4 histone acetyltransferase complexes, acts as a global regulator with growth-coupled effects on stress tolerance and xylose metabolism. Deletion of *TRA1* significantly reduced the final biomass in xylose medium. Moreover, deletion of *RTT109* specifically impaired growth on xylose without affecting any of the tested stress tolerance phenotypes. We further examined global chromatin accessibility changes upon deletion of the histone acetyltransferase gene *NGG1*, a manipulation previously shown to substantially enhance xylose utilization in the engineered YB-2625 background. ATAC-seq analysis revealed that loss of Ngg1 alters chromatin accessibility at loci governing carbohydrate metabolism and stress responses, thereby establishing a direct link between epigenetic remodeling and the superior phenotype of YB-2625. Our findings provide a basis for deciphering the regulatory circuitry governing xylose utilization in recombinant yeast and for the rational engineering of robust strains for lignocellulosic bioconversion.

## 1. Introduction

The transition from fossil-based to renewable resources has rendered lignocellulosic biomass a key resource for sustainable fuel and chemical production [1]. Second-generation bioethanol, derived from agricultural residues and energy crops, offers a promising strategy to reduce greenhouse gas emissions and enhance energy security [2]. However, the economic viability of this process is severely constrained by the inability of conventional ethanologenic yeasts to ferment all of the sugars present in lignocellulosic hydrolysates. Although *S. cerevisiae* readily converts glucose, its poor utilization of D-xylose, the second most abundant sugar, remains a major bottleneck [3]. This limitation arises from several intrinsic barriers, such as the lack of a specific xylose transporter, which forces xylose uptake to rely on native hexose transporters with low affinity and strong glucose repression, and the absence of an endogenous xylose isomerization pathway, which requires xylose to be converted to xylulose via heterologous routes before entering central metabolism.

Extensive efforts have been devoted to engineering *S. cerevisiae* for xylose fermentation through heterologous pathways, such as the xylose reductase/xylitol dehydrogenase (XR-XDH) or xylose isomerase (XI) routes [4]. Numerous studies have focused on engineering xylose transport, including overexpression or directed evolution of endogenous transporters and the introduction of heterologous xylose transporters from other yeasts [5,6]. Despite these advances, engineered strains frequently exhibit slow xylose consumption, cofactor imbalance, and undesirable byproduct accumulation [7,8]. Crucially, the regulatory networks that coordinate xylose assimilation with stress defense and growth control remain poorly understood, especially those governing epigenetic remodeling and chromatin accessibility. This limited understanding has constrained our ability to rationally rewire cellular metabolism for robust xylose fermentation [4,9].

An alternative and complementary strategy is to exploit naturally xylose-fermenting isolates of *S. cerevisiae* [10]. Population genomics has revealed that wild strains harbor extensive genetic diversity in subtelomeric regions and transposable elements, especially in genes involved in chromatin organization, transcriptional regulation, and stress signaling [11,12,13]. Such natural variants may harbor regulatory or structural mutations that have been evolutionarily optimized for xylose utilization without extensive heterologous gene expression [10]. Notably, exploring natural yeast diversity to uncover endogenous regulatory mechanisms is emerging as a powerful complement to classical metabolic pathway engineering [14] and the rational exploitation of yeast chassis backgrounds [15,16]. Indeed, comparative studies have revealed that variations in histone modifiers, ATP-dependent remodelers, and transcription factors can profoundly alter carbon metabolism and stress tolerance [9,17,18,19]. Nevertheless, the specific molecular mechanisms by which these epigenetic and regulatory variations confer superior phenotypes in most natural isolates remain largely elusive. Recent studies have demonstrated that systematic exploration of natural yeast diversity, combined with multi-omics profiling and targeted engineering, provides a powerful framework for developing robust industrial cell factories [15,16,20].

We previously reported that *S. cerevisiae* YB-2625, isolated from sugarcane bagasse, exhibits superior xylose-fermenting capacity relative to laboratory strains [21]. Transcriptomic comparisons between YB-2625 and the reference strain S288C revealed enhanced expression of genes involved in xylose assimilation, gluconeogenesis, and oxidative stress defense. YB-2625 exhibits enhanced transcription of multiple hexose transporters with xylose transport capacity, including *HXT3*, *HXT4*, *HXT5*, *HXT7*, and *HXT13*, compared with the reference strain S288C [21]. Furthermore, YB-2625 shows alleviated glucose repression, with reduced transcription of *MIG1*, *MIG2*, *MIG3*, and *HXK2*, which may facilitate xylose uptake through de-repression of *HXT* transporters even in the presence of glucose. Notably, deletion or mutation of *HXK2* has been shown to improve glucose-xylose co-consumption in engineered *S. cerevisiae* strains [14]. In addition, YB-2625 also displays increased expression of endogenous xylose-metabolizing genes, including the xylitol dehydrogenase gene *XYL2*, the xylulokinase gene *XKS1*, and multiple aldo-keto reductase genes *GRE3*, *GCY1*, and *YPR1* that are potentially involved in xylose reduction [21]. The potential of this natural isolate as a robust platform for lignocellulosic bioconversion is further supported by the development of a recombinant strain with tolerance to inhibitors in lignocellulosic hydrolysates [22]. However, the genetic and epigenetic determinants underlying these remarkable phenotypes had not been identified.

In this study, we performed an integrated genomic and transcriptomic analysis of YB-2625 to elucidate the regulatory rewiring that underpins its efficient xylose utilization and stress tolerance. Comparative genomic analysis with S288C revealed genetic variations significantly enriched in genes involved in chromatin remodeling, transcriptional regulation, and stress signaling. Integration of genomic and transcriptomic data identified candidate variants in key regulators, which were subsequently validated through targeted gene manipulation. These findings reveal the effects of natural genetic variation on carbon metabolism and stress tolerance and provide a basis for engineering robust yeast strains for bioproduction using renewable biomass.

## 2. Materials and Methods

### 2.1. Strains and Culture Conditions

*Escherichia coli DH5α* was used for plasmid maintenance and propagation and was cultured in Luria–Bertani (LB) medium, with 100 μg/mL Ampicillin added when necessary. All yeast strains used in this study are listed in Table S1. Specifically, the host strain used for genetic manipulation in this study was YRH396h, a haploid derivative of the engineered xylose-utilizing strain YRH396 using the strain *S. cerevisiae* YB-2625 as the parent strain [17,23]. YRH396 was originally constructed by integrating the *Scheffersomyces stipitis XYL1*, *XYL2*, and *S. cerevisiae XKS1* genes into the genome of the natural isolate YB-2625 [23], and its haploid derivative YRH396h was subsequently obtained by sporulation [17]. YRH396h retains the YB-2625 genetic background but is haploid and harbors the heterologous xylose assimilation pathway. YPD medium, consisting of 10 g/L yeast extract, 20 g/L peptone, and 20 g/L glucose, was used for yeast cell cultivation, and 20 g/L agar was added to prepare solid media. For selection of transformants, 100 μg/mL nourseothricin, 300 μg/mL G418 disulfate, or 300 μg/mL hygromycin B were added as appropriate. YPX40 medium composed of 4 g/L yeast extract, 3 g/L peptone, and 40 g/L xylose was used for xylose fermentation. After seed culture in YPD medium, the cells were inoculated at 10% (*v*/*v*) into 250 mL flasks containing 100 mL YPX40, and fermentation was performed at 30 °C, 150 rpm under micro-aerobic conditions. YPDX5, consisting of YPD supplemented with 5.0 g/L xylose, was used to evaluate the xylose utilization capacity of various wild-type *S. cerevisiae* strains.

### 2.2. DNA Preparation, Genome Sequencing and Analysis

Genomic DNA from *S. cerevisiae* YB-2625 was isolated from yeast cells grown overnight in YPD medium, and its quality and quantity were determined using a NanoDrop 2000 spectrophotometer (Thermo Fisher Scientific, Waltham, MA, USA). For library preparation, the extracted genomic DNA was randomly fragmented, and DNA fragments of the desired length were recovered by gel electrophoresis. A sequencing library was then constructed and sequenced on an Illumina HiSeq 2500 platform (Shanghai Oebiotech Co., Ltd., Shanghai, China), following a standard protocol. The sequencing produced a total of 12,868,910 reads with an average read length of 125 bp, generating 2 GB of raw data, which corresponds to more than 150-fold coverage of the *S. cerevisiae* genome. Raw reads were quality filtered using NGSQC (v0.31) and clean reads were aligned to the *S. cerevisiae* S288C reference genome with BWA-MEM (0.7.12). Variant analysis was conducted using SAMtools (1.2), and structural variants (SVs) were detected using Pindel (0.2.5). Single nucleotide variants and small insertions/deletions were further annotated using SnpEff (version 4.1g) with S288C as the reference. The genome assembly for YB-2625 has been deposited in the NCBI database under BioProject accession number PRJNA1492256. To validate the accuracy of variant calling, five genes carrying different types of mutations were independently amplified by PCR from YB-2625 genomic DNA using the primers listed in Table S2, and the resulting PCR products were sequenced.

### 2.3. Stress Tolerance Assay

Stress tolerance of yeast strains was evaluated using a spot assay. Yeast cells were cultured in YPD medium at 30 °C for 24 h and then transferred to fresh YPD medium and cultured to the stationary phase. The pellets of yeast strains with flocculent ability were first deflocculated by 0.1 M sodium citrate. The OD_600_ of the broth was adjusted to the same level with distilled water, and 2.0 μL of the ten-fold diluted suspensions of each strain was spotted on solid YPD medium supplemented with 5 mM hydrogen peroxide, 5 g/L acetic acid, or 10% ethanol. YPD medium without inhibitor supplementation served as the control. The plates were incubated at 30 °C for 2 days, while solid YPD medium incubated at 40 °C were used for evaluating the thermotolerance ability of the different yeast strains.

### 2.4. Construction of Gene Deletion and Overexpression Mutants

All genetic manipulations were performed in the host strain YRH396h using the lithium acetate transformation method [24]. For gene deletion, knockout cassettes containing the *HphMX* marker and 40 bp homology arms to the target ORF were amplified from pRS41H using specific primers (Table S3). Transformants were selected on YPD agar containing 300 μg/mL hygromycin B, and positive integration was confirmed by PCR using external and internal marker-specific primers.

For gene overexpression, the ORFs of *NGG1*, *RTT109*, *PRX1*, *CTT1*, and *SSB2* were amplified from genomic DNA of YB-2625 or S288C using gene-targeted primers (Table S3) and cloned into the *PGK1* promoter-driven expression vector pRS41H-*PGK1p*-*yEGFP* with hygromycin B resistance marker. Positive transformants were verified by PCR using a promoter-specific forward primer and a gene-specific reverse primer. The empty vector was used as a negative control, and two independent transformants per construct were used for subsequent analyses.

### 2.5. RNA-Seq and ATAC-Seq Analyses

The RNA-seq dataset for YB-2625 and S288C was obtained from our previous study [21], in which both strains were cultivated in YPD80X20 medium, consisting of 4 g/L yeast extract, 3 g/L peptone, 80 g/L glucose, and 20 g/L xylose at 30 °C and 150 rpm under micro-aerobic conditions. Cells were harvested at 7 h during the mixed-sugar stage and at 48 h during the xylose-only stage after glucose depletion for Illumina NovaSeq 6000 sequencing (Illumina, Inc., San Diego, CA, USA). For ATAC-seq, nuclei were extracted from cells harvested at mid-exponential phase in YPX40 medium, and 40,000 nuclei per sample were incubated with Tn5 transposase at 37 °C for 30 min, following established protocols [18,25]. Tagmented DNA was purified, amplified, and sequenced on an Illumina NovaSeq 6000 platform (Illumina, Inc., San Diego, CA, USA) to generate 150-bp paired-end reads. Three independent biological replicates were analyzed per strain. Raw reads were trimmed using Trimmomatic (v0.39) and aligned to the S288C reference genome using BWA-MEM (0.7.17). Peak calling was performed with MACS2 (2.2.9.1) (q < 0.05), and differential accessibility between the Δ*NGG1* mutant and wild-type YRH396h was analyzed using DESeq2 (1.48.1) with thresholds of |FoldChange| > 1.5 and *p* < 0.05. Differentially accessible regions were annotated to nearby genes using HOMER (v4.11), and functional enrichment was analyzed using clusterProfiler (4.19.6). The comparative RNA-seq datasets for YB-2625 and S288C, and ATAC-seq datasets for the Δ*NGG1* mutant have been deposited under BioProject PRJNA1514219 and PRJNA1497054, respectively.

### 2.6. Analytical Methods

Cell growth was monitored by OD_600_ measurements using a Multiscan GO microplate reader (Thermo Fisher Scientific, Waltham, MA, USA). All samples were diluted to ensure OD_600_ readings within the linear range of 0.1–1.0. The final OD_600_ values were calculated by multiplying the measured values for the diluted samples by the corresponding dilution factor. The samples were centrifuged at 10,000× *g* for 3 min, and the supernatants were filtered through a 0.22 μm membrane. Concentrations of ethanol in fermentation broth were determined by high-performance liquid chromatography (HPLC) (Waters 410, Waters Corporation, Milford, MA, USA) equipped with the Aminex HPX-87H column (300 mm × 7.8 mm, Bio-Rad, Hercules, CA, USA) following the previously described protocol [26].

### 2.7. Statistical Analysis

All experiments were performed with three biological replicates. Quantitative data were presented as the mean value ± standard deviation (SD). Statistical analysis was performed using Student’s *t*-test, with statistical significance set at *p* < 0.05 and *p* < 0.01, respectively.

## 3. Results and Discussion

### 3.1. Superior Growth and Stress Tolerance of YB-2625

**Figure 1:**
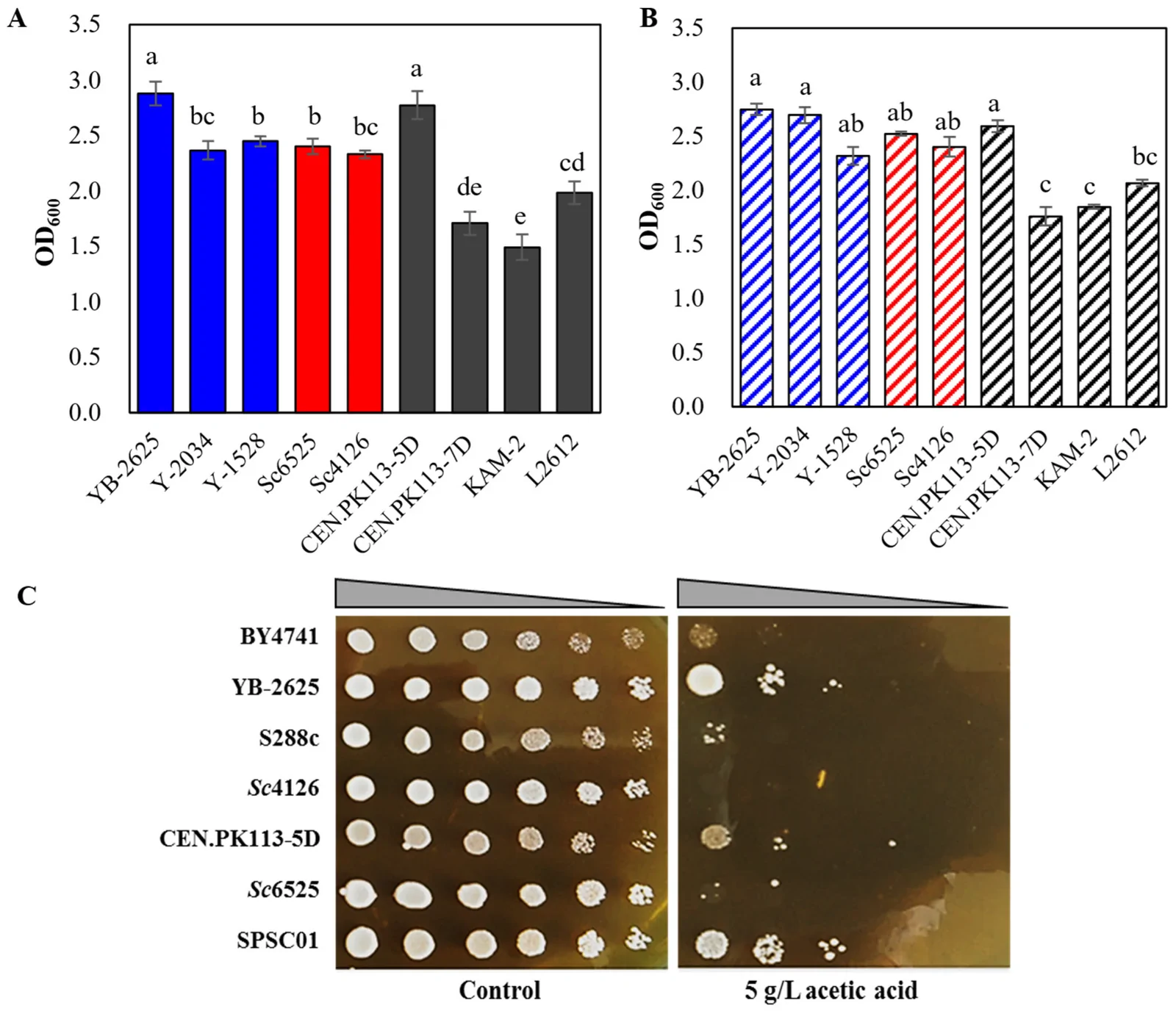
Comparison of carbon sources metabolism and stress tolerance among different *S. cerevisiae* strains. (**A**,**B**), Growth of yeast strains in YPD and YPDX5 media. Blue, red, and black bars denote natural, industrial, and laboratory yeast strains, respectively. Growth was determined in 250 mL Erlenmeyer flasks at 30 °C, 150 rpm under micro-aerobic conditions for 12 h. Data are presented as the mean ± standard error of the mean (SEM) of biological replicates. One-way ANOVA followed by Tukey’s HSD post hoc test was used for multiple pairwise comparisons between strains, without designating a single reference control. Columns denoted by different lowercase letters differ significantly (*p* < 0.05), while those sharing the same letter are not significantly different. (**C**), Acetic acid tolerance of yeast strains. Cell growth was evaluated on YPD agar medium supplemented with 5 g/L acetic acid using a 10-fold dilution at 30 °C, and YPD medium without added acetic acid was used as the control. All experiments were performed in triplicate with reproducible results and representative images are shown.

To evaluate the potential of the natural isolate YB-2625 as a robust chassis, its growth and stress tolerance were compared with those of other *S. cerevisiae* strains (Table S1). YB-2625 exhibited a growth advantage in YPD and YPDX5 media (Figure 1A,B). Moreover, YB-2625 displayed substantially more robust and sustained xylose consumption than S288C in mixed-sugar fermentation with 80 g/L glucose and 20 g/L xylose, consuming 15.2 g/L xylose within 96 h compared with only 7.6 g/L consumed by S288C [21]. This superior performance, occurring after glucose depletion, reflects a sequential utilization pattern and highlights the enhanced capacity of YB-2625 for xylose metabolism upon relief of glucose repression. Furthermore, YB-2625 displayed superior tolerance to 5.0 g/L acetic acid and a high temperature of 40 °C (Figure 1C and Figure S1), consistent with its potential as a robust host for lignocellulosic bioethanol production [27,28]. Notably, YB-2625 exhibits inherently superior growth even on inhibitor-free YPD medium, reflecting enhanced intrinsic energy balance. This underlying metabolic fitness likely serves as an energetic foundation, enabling the cells to better mobilize specific defense mechanisms when exposed to environmental stresses. Therefore, the cooperative interplay between its robust basal metabolism and specialized stress responses confers superior overall tolerance of acetic acid and heat stress on YB-2625 [9,27]. These phenotypic features, particularly the glucose-dependent xylose utilization advantage and enhanced stress tolerance, prompted further investigation of the underlying genomic and regulatory basis.

### 3.2. Genomic Variations Associated with the Robust Phenotype of YB-2625

To identify the genetic basis underlying glucose-dependent xylose utilization and stress tolerance of YB-2625, whole-genome sequencing was performed. The genome assembly covered 97.4% of the reference genome and contained 5507 predicted coding sequences. Relative to S288C, YB-2625 harbored 73,842 single-nucleotide polymorphisms (SNPs) and 5191 small insertions/deletions (InDels), with significant enrichment in genes involved in chromatin organization and transcriptional regulation (Figure S2). The reliability of variant identification was confirmed by independent PCR and sequencing of five representative genes (Table S2).

In addition to point mutations, prominent structural variations included tandem duplications (TD) within the *FLO* gene family, namely *FLO1*, *FLO5*, *FLO9*, *FLO10*, and *FLO11*, as well as large-scale inversions in the mitochondrial genome. These *FLO* duplications, together with in-frame insertions in the flocculation regulator *FLO8*, underpin the strong flocculation phenotype of the YB-2625-derived xylose-fermenting recombinant strain, enabling rapid sedimentation within 2 min. As reported, mutation of *FLO8* directly contributes to the flocculent phenotype of yeast strains [29]. Flocculation properties are known to enhance acetic acid tolerance [30], aligning with the higher resistance of YB-2625 (Figure 1C). Such genomic features likely confer adaptive advantages under the fluctuating nutrient and stress conditions typical of the bioconversion of lignocellulosic biomass, especially under conditions characterized by episodic nutrient influxes and high acetic acid concentrations, such as those encountered in the sugarcane bagasse habitat from which YB-2625 was isolated. In addition, frameshift and stop-gained mutations resulting from TDs were detected in the hexose transporter genes *HXT3* and *HXT6*, while non-synonymous SNPs were found in the glucose repressor genes *MIG1* and *MIG3*. Deletion of *MIG1* has been shown to partially relieve glucose repression [31]. These genetic alterations may contribute to the alleviated glucose repression phenotype previously observed in YB-2625 [21].

Moreover, we detected 57 mitochondrial inversions (Figure S3A) in oxidative phosphorylation genes such as *COX2*, *COX3*, *COB*, and *OLI1*, as well as genes encoding mitochondrial ribosomal proteins and tRNAs. Considering their locations in core respiratory genes, these inversions are predicted to impair respiratory chain function. To provide direct physiological evidence for this prediction, a TTC reduction assay was performed (Figure S3B), and the results revealed significantly reduced but not completely abolished respiratory capacity in YB-2625 compared to the control strain S288C. This phenotypic observation aligns well with the predicted impairment of respiratory chain function caused by these mitochondrial inversions. Given that reduced respiration under micro-aerobic conditions has been previously linked to the redirection of carbon flux toward the pentose phosphate pathway (PPP) and enhanced xylose utilization [32], these findings support the proposed metabolic rewiring. Nevertheless, direct verification of carbon metabolic flux redistribution via metabolic flux analysis remains necessary to definitively confirm this hypothesis. Intriguingly, YB-2625 maintained intracellular ROS levels that were 43.3% and 58.6% lower than S288C at the mixed-sugar and xylose-only stages, respectively, [21] despite these mitochondrial perturbations, implying that its superior xylose fermentation is underpinned by both metabolic rewiring and efficient oxidative stress defense.

These genomic features, particularly the enrichment of variations in chromatin regulators, mitochondrial function, and carbon metabolism, point to a multilayered regulatory network underlying the phenotype of YB-2625. To explore the functional relevance of these variations, genomic and transcriptomic data were integrated, followed by targeted genetic validation of selected candidate genes.

### 3.3. Epigenetic and Transcriptional Regulatory Nodes Identified by Integrated Omics

**Figure 2:**
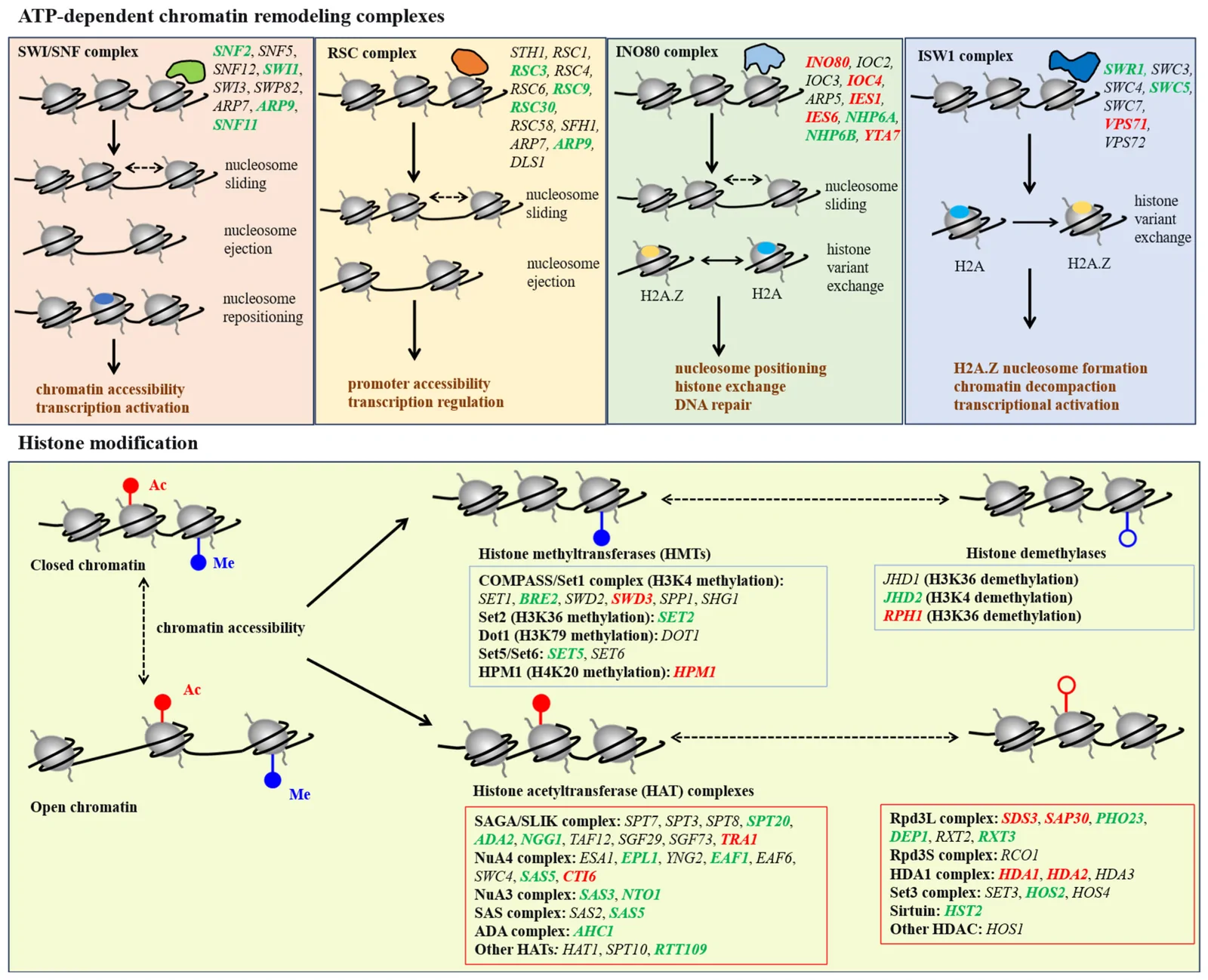
Schematic overview of chromatin regulatory complexes with mutations or differential expression in YB-2625. All genes shown in this schematic carry non-synonymous SNPs or InDels in the YB-2625 genome relative to the reference strain S288C. Genes highlighted in green are significantly down-regulated (|log_2_FC| ≥ 0.5, *p* < 0.05) at either the mixed-sugar stage (7 h) or the xylose-only stage (48 h), while genes in red are significantly up-regulated at either stage. The schematic covers histone acetyltransferases, deacetylases, methyltransferases, demethylases, and ATP-dependent remodelers. Dashed double headed arrows indicate nucleosome sliding, reversible chromatin state transitions and histone modification cycles, including acetylation deacetylation and methylation demethylation. Red and blue dots represent histone acetylation and methylation, respectively.

To identify candidate regulators for functional validation, genomic variations were integrated with transcriptomic profiling of YB-2625 and S288C during mixed-sugar and xylose-only fermentation [21]. This integration revealed that the majority of mutated chromatin regulatory genes were differentially expressed in YB-2625 relative to S288C (Figure 2), suggesting a broadly altered epigenetic landscape.

YB-2625 harbors mutations in multiple classes of chromatin regulators, including ATP-dependent remodelers, histone methyltransferases, demethylases, and acetyltransferase-associated regulators (Figure 2). Among ATP dependent remodelers, *INO80* was up-regulated during the mixed-sugar stage, while *SWR1* was down-regulated specifically during xylose fermentation. Overexpression of *INO80* enhances stress tolerance and non-preferred nitrogen utilization under acetic acid stress [18], whereas *SWR1* overexpression increases xylose utilization by modulating H2A.Z occupancy at carbon regulators [33]. In YB-2625, the down regulation of *SWR1* during xylose utilization may represent a strain-specific adaptation that redirects carbon flux, while the transient up regulation of *INO80* likely reinforces stress defense under mixed-sugar conditions. Variations were also detected in histone methyltransferases *HPM1*, *SET2*, *BRE2*, *SET5* and demethylases *JHD2* and *RPH1*. Notably, *SET5*, whose overexpression improves acetic acid tolerance through Hog1 pathway activation [34], is down-regulated in YB-2625, potentially reflecting an energy-conserving adaptation to the lower basal oxidative stress [21]. In contrast, *RPH1*, an activator of acetate and glycerol biosynthetic genes under glucose-limited conditions [35], is up-regulated during xylose-only fermentation. Nevertheless, YB-2625 accumulates fewer byproducts than S288C despite *RPH1* up-regulation, pointing to a decoupling between Rph1-dependent transcription and its metabolic consequences, potentially via mutations in target genes or post transcriptional modulation. In addition, mutations and expression changes were noted in the chromatin regulator-encoding genes *RTT109*, *NGG1*, and *TRA1*, which were selected for subsequent functional validation.

Besides the chromatin-related alterations described above, mutations and expression changes were observed in stress-responsive transcription factors, including the oxidative stress activator *YAP1* and the general stress regulators *MSN2/MSN4*. For nitrogen metabolism, mutations were found in *GAT1* and *GLN3*, activators of nitrogen catabolite repression, as well as in *GCN4*, a master regulator of amino acid biosynthesis under nutrient limitation [36,37]. These findings suggest that the carbon–nitrogen balance is a critical aspect of the regulatory rewiring in YB-2625. More notably, the ribosome-associated Hsp70 chaperone *SSB2*, which is expressed in YB-2625 but absent in S288C, was identified as a strain-specific element potentially involved in protein folding during xylose metabolism. Ssb has been shown to associate with Snf1 and regulate glucose signaling [38], suggesting a potential link between chaperone function and carbon metabolism. These transcriptomic and genomic data identify *SSB2* as a non-intuitive engineering target, which was subsequently tested by overexpression. Efficient xylose fermentation relies on regulatory rewiring of global signaling networks that coordinate growth, metabolism, and stress defense [9], and the pervasive variations in chromatin and stress regulators in YB-2625 suggest that this natural isolate may have experienced such rewiring through evolutionary adaptation.

### 3.4. Functional Validation of Candidate Genes via Genetic Manipulation

To directly assess the functional relevance of the genomic and transcriptomic variations identified in YB-2625, targeted gene knockouts and overexpression experiments were performed in the recombinant xylose-utilizing haploid strain YRH396h. To prioritize candidate genes among the extensive variants in YB-2625, an integrated multi-omics filter was applied to genes that harbored non-synonymous SNPs or frame-shift InDels, exhibited significant differential expression during xylose fermentation, and encoded regulatory nodes within chromatin remodeling or stress response networks. Accordingly, the selected candidates included the chromatin modifiers *NGG1*, *TRA1*, and *RTT109*, the signaling regulator *SKO1*, the uncharacterized gene *YGR067C*, and the stress-protective chaperone *SSB2* (Table S4).

#### 3.4.1. Phenotypic Effects of Gene Knockouts on Stress Tolerance and Xylose Metabolism

**Figure 3:**
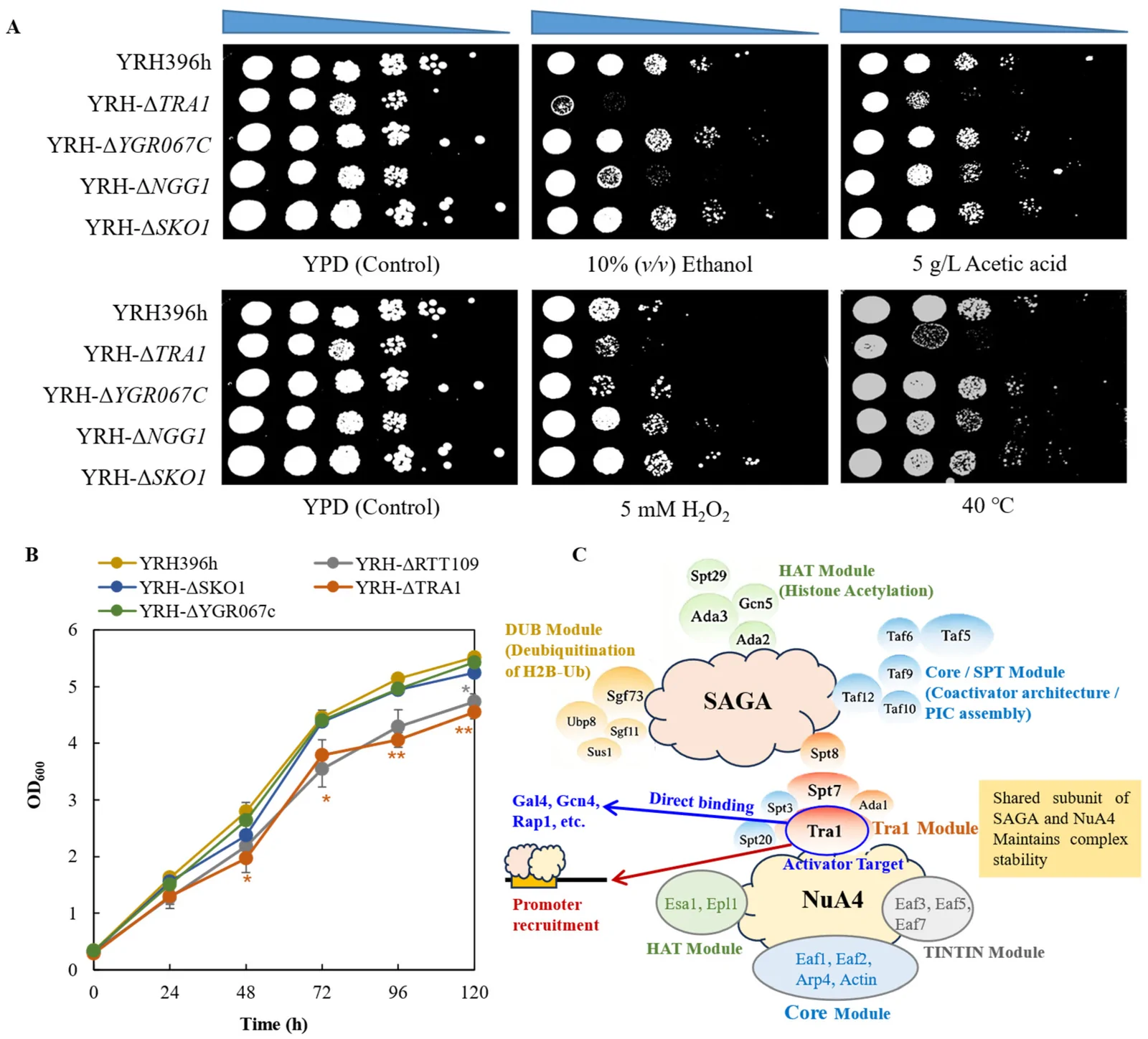
Stress tolerance and growth performance of wild-type and gene-knockout yeast strains. (**A**), Spot dilution growth assays of the wild-type strain YRH396h and its knockout derivatives YRH-Δ*TRA1*, YRH-Δ*YGR067C*, YRH-Δ*SKO1*, and YRH-Δ*RTT109* under various stress conditions. Serial dilutions of each strain were spotted onto YPD medium (control) or YPD medium supplemented with 10% (*v*/*v*) ethanol, 5 g/L acetic acid, 5 mM H_2_O_2_, or were incubated at 40 °C. Plates were photographed after incubation to assess growth phenotypes. (**B**), Growth curves of the yeast strains over 120 h of cultivation in medium containing 40 g/L xylose. Cell growth was monitored by measuring the optical density at 600 nm (OD_600_) at 24 h intervals. Data points represent the mean ± standard deviation of biological replicates. Significant differences between mutants and the control at each time point are denoted by asterisks: * *p* < 0.05, ** *p* < 0.01. Asterisks are color-matched to the corresponding curves. (**C**), Schematic representation of the SAGA and NuA4 coactivator complexes highlighting the shared subunit Tra1.

The stress tolerance of the control strain YRH396h and its knockout derivatives was evaluated using spot assays, while their xylose utilization capacity was assessed using growth curves in xylose-containing medium (Figure 3 and Figure S4).

Deletion of *TRA1*, which encodes a shared subunit of the SAGA and NuA4 histone acetyltransferase complexes [39,40,41], slightly impaired growth under non-stress conditions but severely compromised growth under all tested stress conditions, including 10% ethanol, 5 g/L acetic acid, 5 mM H_2_O_2_, and 40 °C (Figure 3A). The Δ*TRA1* mutant also exhibited significantly reduced biomass accumulation in xylose medium, with a final OD_600_ of 4.55 after 120 h, compared with 5.51 for the control (Figure 3B). As shown in Figure 3C, Tra1 functions as a scaffold that maintains the structural integrity of both SAGA and NuA4 and mediates the recruitment of transcriptional activators such as Gal4, Gcn4, and Rap1 [40,41]. In addition, Tra1 is required for proper NuA4-mediated H4 acetylation at target promoters, including the promoter of *PMA1*, a key H^+^-ATPase involved in membrane homeostasis [42]. In addition to its role in transcription, Tra1 coordinates mitochondrial function and membrane trafficking [43], and its essential role is further underscored by a recent CRISPRi screen linking *TRA1* repression to increased acetic acid sensitivity [44]. In addition to its roles in stress responses in laboratory strains, the present study demonstrates that Tra1 regulates both general stress tolerance and xylose metabolism in a background derived from a natural isolate, highlighting its function as a coactivator node that integrates chromatin remodeling, carbon metabolism, and stress defense.

In contrast, deletion of *RTT109* did not affect stress tolerance (Figure S4) but significantly impaired growth on xylose (Figure 3B), suggesting a specific role in carbon metabolism rather than stress defense. Rtt109 is a histone H3 acetyltransferase responsible for H3K56 acetylation, which is involved in DNA replication and repair [45]. Its requirement for xylose metabolism may reflect the need for proper chromatin dynamics during metabolic adaptation to non-fermentable carbon sources. Interestingly, *RTT109* deletion in the BY4741 background was previously shown to improve acetic acid tolerance [46], whereas no such effect was observed in the YB-2625-derived background, highlighting the influence of host genetic context on phenotypic effects [44].

Deletion of *SKO1*, which encodes a bZIP transcription factor downstream of the HOG1 pathway, enhanced tolerance to 5 mM H_2_O_2_ (Figure 3A). Deletion of *SKO1* eliminates recruitment of the Cyc8-Tup1 corepressor, thereby derepressing oxidative defense genes such as *GRE2* and *AHP1* and increasing H_2_O_2_ tolerance [47]. The Δ*SKO1* mutant also exhibited thermo-sensitivity at 40 °C, indicating a broader role for Sko1 in heat shock or cell wall integrity pathways, as reported in *Candida albicans* [48]. However, *SKO1* deletion had no effect on growth on xylose (Figure 3B), confirming its primary role in stress signaling rather than carbon metabolism. *YGR067C* encodes a putative zinc-finger protein of unknown function. In the BY4741 background, its deletion downregulates ethanol-consuming respiratory pathways during the diauxic shift without affecting fermentative growth on glucose [49]. In addition, deletion of *YGR067C* specifically reduced tolerance to hydrogen peroxide without affecting other stresses or xylose utilization in the YB-2625-derived YRH396h background (Figure 3). This observation, together with its up-regulation during both the mixed-sugar and xylose stages in YB-2625, suggests a specialized role in oxidative stress defense that may contribute to the lower intracellular ROS levels in this strain [21].

Collectively, these results demonstrate that natural variation in epigenetic regulators and stress responsive transcription factors can influence carbon metabolism and stress tolerance in a background dependent manner, underscoring the value of natural isolates for uncovering non-intuitive engineering targets. The observed biomass accumulation on xylose supports the notion that xylose catabolism contributes to cellular ATP supply in this strain. Based on the observed phenotypic outcomes, *TRA1* was identified as a global regulator associated with growth-coupled defects. *RTT109* specifically modulated xylose metabolism, whereas *SKO1* specifically contributed to oxidative stress defense. Notably, deletion of *NGG1* has previously been reported to enhance xylose utilization and contribute to oxidative stress defense [21], while not affecting general growth fitness. In the current study, xylose-based shake-flask fermentation serves as an initial platform for target discovery via multi-omics screening, while YPD provides a reliable baseline for evaluating intrinsic growth and stress tolerance. However, it should be noted that the stress tolerance assays in this study were performed using glucose as the sole carbon source. Future studies will incorporate both tolerance assays and fermentation performance evaluations using xylose, high-concentration glucose, mixed-sugar media, or lignocellulosic hydrolysates to better understand the interplay between carbon metabolism and stress responses and to ultimately validate the industrial applicability of these engineered strains. In addition to loss-of-function analyses, we next examined whether allele-specific sequence variations in these epigenetic regulators confer functional differences.

#### 3.4.2. Allele Specific Overexpression of *RTT109* and *NGG1* Reveals Context-Dependent Epigenetic Regulation

**Figure 4:**
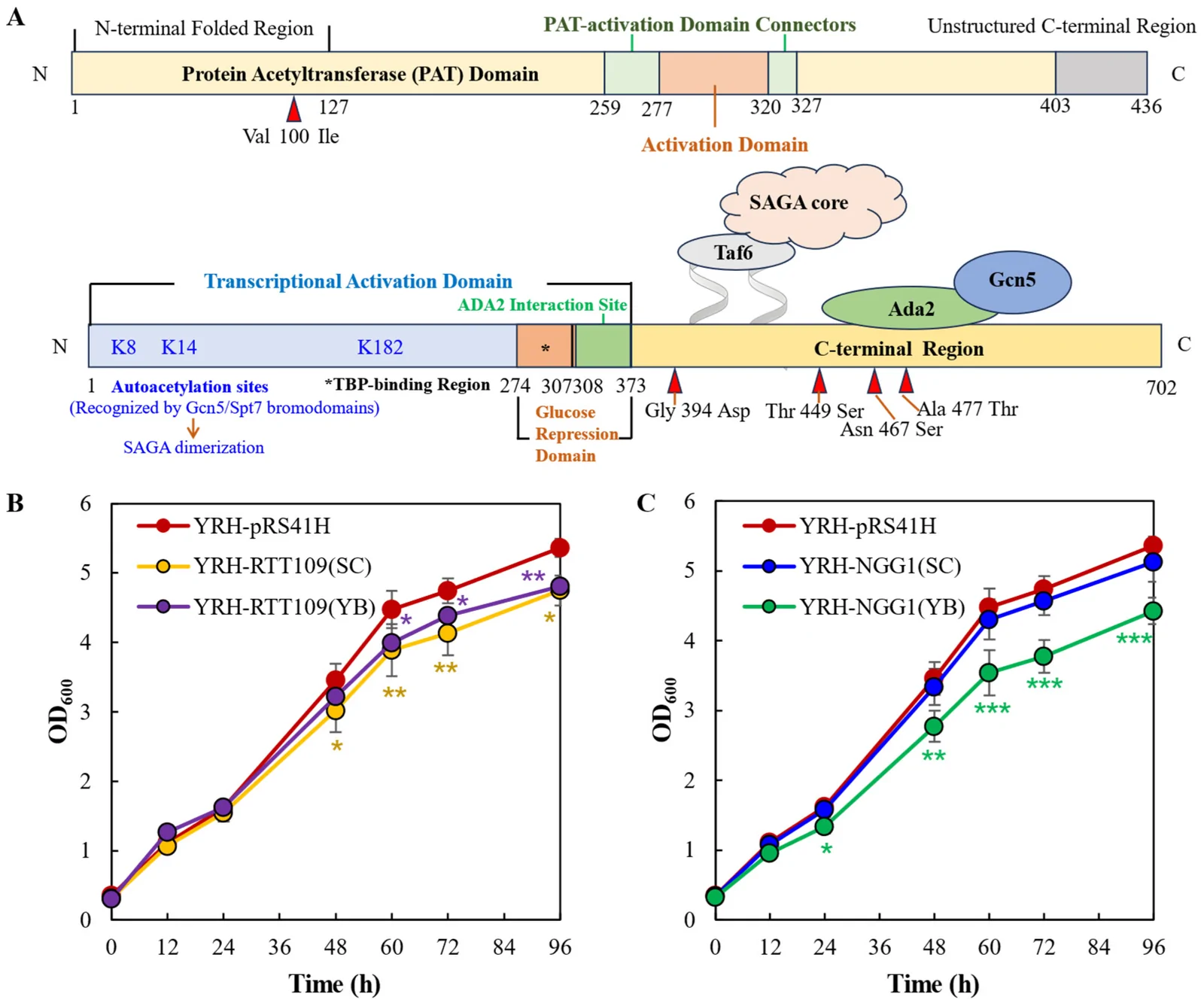
Domain architecture of Rtt109 and Ngg1 proteins, and growth kinetics of yeast strains overexpressing *RTT109* and *NGG1* alleles derived from S288C and YB-2625, respectively. (**A**), Linear domain diagrams of full-length Rtt109 and Ngg1 polypeptides. N and C denote the amino-terminal and carboxyl-terminal of the protein, respectively. Red arrowheads indicate amino acid substitution sites unique to YB-2625 relative to S288C. Key functional modules, post-translational modification sites, and protein-protein interaction interfaces, including SAGA complex-binding regions, are annotated for both proteins. (**B**,**C**), Growth profiles of YRH396h strains overexpressing S288C-type (SC) and YB-2625-type (YB) alleles of *RTT109* and *NGG1*, with the empty vector strain YRH-pRS41H as the control. Cell density was monitored by measuring OD_600_ over 96 h of batch cultivation in medium containing 40 g/L xylose. All data are presented as the mean ± SD of three biological replicates. Statistical significance between each overexpression strain and the empty-vector control at each time point is indicated by asterisks: *, *p* < 0.05; **, *p* < 0.01; ***, *p* < 0.001. Asterisks are color-matched to the corresponding curves.

As illustrated in the domain architecture schematics of Rtt109 and Ngg1, the YB-2625 specific missense mutations in Rtt109 and Ngg1 are located in the protein acetyltransferase (PAT) catalytic domain [50] and the C-terminal SAGA-interacting region, respectively (Figure 4A). To assess the functional impact of natural sequence variations in *RTT109* and *NGG1*, the S288C and YB-2625 alleles of the two genes were overexpressed in YRH396h, and growth was monitored in xylose medium (Figure 4B,C).

Overexpression of either *RTT109* allele reduced growth compared with the empty-vector control (Figure 4B). Together with the observation that *RTT109* deletion also impairs growth on xylose (Figure 3B), this indicates that Rtt109 activity must be tightly regulated. Both loss and excess of this H3K56 acetyltransferase impair carbon metabolism. Moreover, the two *RTT109* alleles caused similar growth defects under xylose fermentation conditions, suggesting that the non-synonymous mutations in the YB-2625 allele do not eliminate the essential function of Rtt109p, although context-dependent fine-tuning cannot be excluded.

The YB-2625-specific missense mutations, including residues 394, 449, 467, and 477, are located within the C-terminal region of Ngg1 [51,52]. The clustering of these mutations in this region suggests they may modulate the function of Ngg1 through alternative mechanisms, such as influencing its interaction with Gcn5 or other components of the ADA/SAGA HAT module, altering its incorporation into distinct multimeric complexes, or affecting protein stability and post-translational modifications [51,52,53]. Consequently, these C-terminal substitutions may fine-tune the HAT activity of the ADA/SAGA complex [54]. Consistent with this notion, overexpression of the YB-2625-*NGG1* allele severely impaired growth in xylose, whereas the S288C allele had no obvious effect (Figure 4C), indicating that these C-terminal substitutions exert a dominant-negative effect when the YB-2625 variant is overproduced. Conversely, deletion of *NGG1* improves xylose utilization [17], suggesting that Ngg1p normally represses xylose related pathways. Importantly, YB-2625 naturally expresses *NGG1* at significantly lower levels than S288C, which likely serves to limit the intracellular concentration of this hyperactive variant and mitigate its deleterious effects. It has been reported that Rtt109, NuA4, and Epl3 are essential for xylose viability and that *GCN5* deletion enhances xylose consumption memory via H4K5 acetylation [55]. These observations, together with the natural downregulation of *NGG1* in YB-2625 and its allele-specific overexpression toxicity, reinforce the notion that SAGA-mediated acetylation and broader acetyltransferase networks cooperate to coordinate xylose adaptation.

The contrasting overexpression phenotypes of *RTT109* and *NGG1* further illustrate the distinct roles of these chromatin regulators in xylose metabolism. Overexpression of *RTT109* from both S288C and YB-2625 caused a similar growth defect. This reinforces the role of Rtt109 as a broadly required, dosage sensitive modifier. In contrast, for *NGG1*, the allelic origin determined the phenotype. Only the YB-2625 allele caused toxicity when overexpressed, highlighting Ngg1 as a pathway specific regulator whose function is subject to allele specific optimization.

#### 3.4.3. ATAC-Seq Reveals That *NGG1* Deletion Remodels Chromatin Accessibility at Metabolic and Stress-Responsive Loci

**Figure 5:**
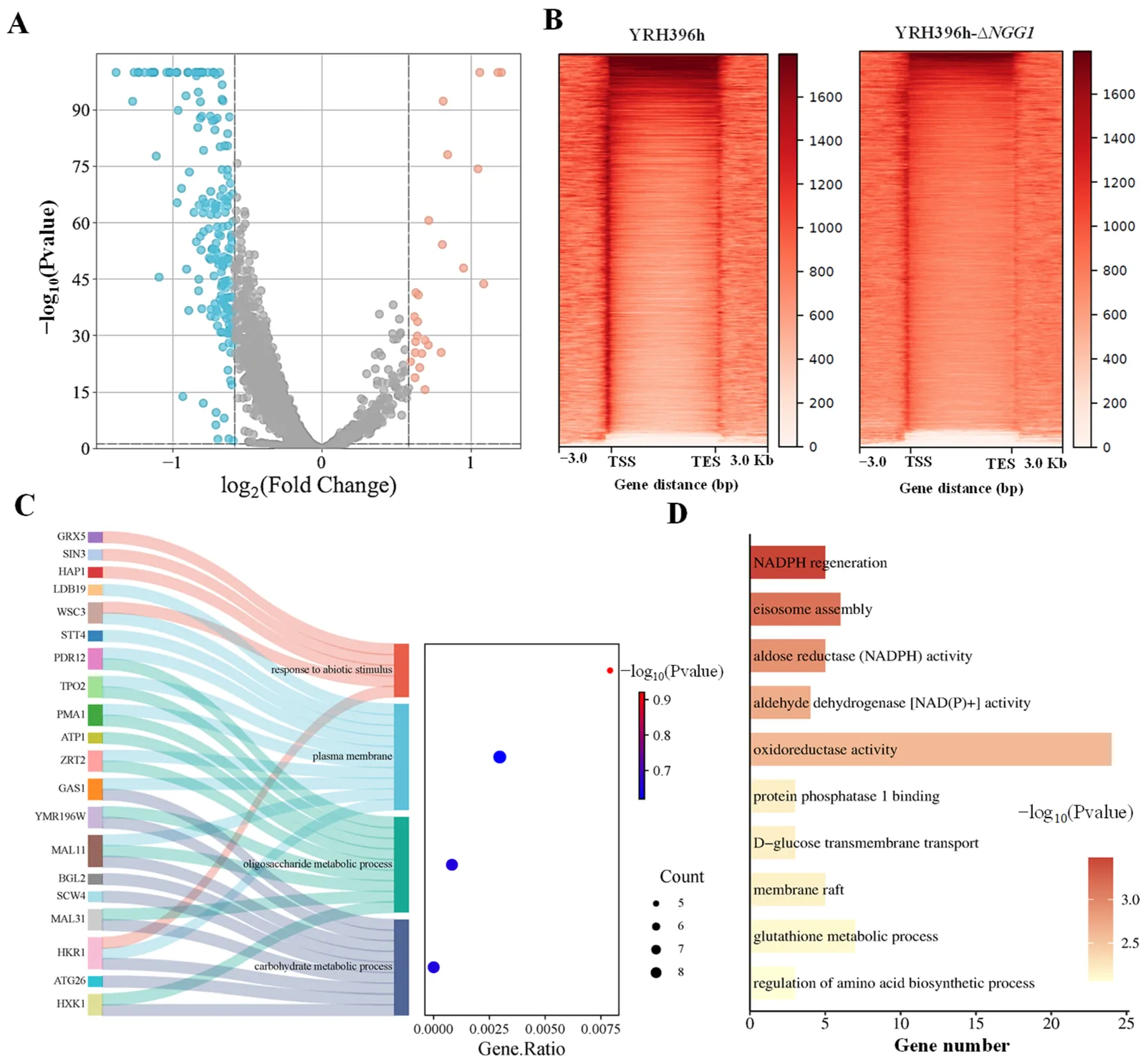
ATAC-Seq analysis reveals altered chromatin accessibility in the Δ*NGG1* mutant. (**A**), Volcano plot of differential chromatin accessibility peaks between YRH396h-Δ*NGG1* and the control strain YRH396h. Red and blue dots indicate significantly upregulated and downregulated peaks, respectively (fold change ≥ 1.5, *p* < 0.05). (**B**), Average ATAC-Seq signal heatmaps over gene bodies from 3 kb upstream of the transcription start site (TSS) to 3 kb downstream of the transcription end site (TES) for both strains. Color intensity represents the mean read signal at each position. (**C**), Bubble plot and Sankey diagram for Gene Ontology (GO) enrichment of genes associated with up-regulated chromatin accessible peaks. (**D**), Bar plot of top enriched GO terms for genes linked to down-regulated peaks.

Deletion of *NGG1* has been shown to improve xylose utilization and oxidative stress tolerance in the YRH396h background [17]. Moreover, *NGG1* is transcriptionally down-regulated in the natural isolate YB-2625 relative to S288C. To explore whether chromatin-level remodeling underlies the benefits of diminished *NGG1* function, we performed ATAC-seq on the Δ*NGG1* mutant during xylose fermentation. Loss of *NGG1* led to 181 regions with reduced chromatin accessibility and 25 regions with increased chromatin accessibility (fold change ≥ 1.5, *p* < 0.05), predominantly near transcription start sites (Figure 5A,B). Genes associated with these differentially accessible regions (DARs) were significantly enriched in functional categories related to carbohydrate metabolism, membrane transport, and stress defense (Figure 5C,D). More specifically, DARs were significantly enriched in genes involved in glucose transmembrane transport, glutathione metabolism, and the regulation of amino acid biosynthetic process.

To identify chromatin changes with direct transcriptional consequences, ATAC-seq data were integrated with transcriptomic profiles from the Δ*NGG1* mutant. Representative genes with concordant changes, defined as increased accessibility coupled with elevated transcription or decreased accessibility coupled with reduced transcription, are listed in Table 1. Genes with increased accessibility were enriched in plasma membrane transport and cell wall organization, including *PMA1* encoding the H^+^ ATPase, *STT4* encoding a phosphatidylinositol 4-kinase, and several genes encoding mannoproteins and glucanosyltransferases, such as *FLO5*, *HSP150*, *GAS1*, *SCW4*.

Conversely, multiple stress-inducible genes exhibited reduced accessibility, as did genes involved in alternative carbon utilization and respiration (Table 1). In the control strain, these stress-related and alternative carbon genes are induced in response to xylose, a non-preferred carbon source. Their reduced chromatin accessibility may thus reflect a transcriptional reprogramming that accompanies the metabolic shift toward fermentation, as evidenced by the down-regulation of respiratory and starvation response genes [17]. Decreased accessibility was also observed at loci encoding *HXT* hexose transporters. Since *NGG1* encodes a key regulator of glucose repression [53], this alteration may contribute to the alleviated glucose repression and enhanced mixed-sugar utilization in YB-2625 [21]. Moreover, concordant changes in chromatin accessibility and transcription were detected at loci encoding other epigenetic regulators, including the histone demethylase *GIS1*, the transcriptional repressor *NRG2*, and the NuA4 subunit *EPL1*, suggesting that *NGG1* deletion may trigger secondary transcriptional effects that amplify the chromatin-level effects.

Overall, these ATAC-seq data, together with the allele-specific overexpression phenotypes, indicate that *NGG1*-mediated chromatin regulation is associated with the enhanced xylose fermentation of YB-2625. Natural down-regulation of *NGG1* in YB-2625 drives a coordinated metabolic and epigenetic reprogramming that likely underlies its superior xylose fermentation and stress tolerance.

In addition to chromatin regulators, we assessed the functional contributions of several non-intuitive candidates identified from the multi-omics comparison. Overexpression of *SSB2*, a strain-specific Hsp70 chaperone present in YB-2625 but absent in S288C, accelerated growth on xylose and increased ethanol production by 21.5%, but the difference was not statistically significant (*p* > 0.05, Figure S5), while stress tolerance was not affected. Based on the amount of consumed xylose, the control and *SSB2*-overexpressing strains achieved ethanol conversion efficiencies of 32.6% and 38.2% of the theoretical maximum, respectively. These moderate yields are common for non-evolved, base-level engineered *S. cerevisiae* strains, which inherently suffer from redox imbalance and carbon flux to xylitol. Achieving higher yields typically requires extensive adaptive evolution and further metabolic rewiring, indicating a clear direction for future improvement of the YRH396h background. Importantly, despite the limitations of this base strain, *SSB2* overexpression enhanced the conversion efficiency by 5.6% compared with the control. Ssb binds cotranslationally to aggregation-prone nascent polypeptides, and loss of *SSB* leads to widespread aggregation of newly synthesized proteins [56]. The improved xylose fermentation efficiency may thus reflect enhanced folding and functional stabilization of xylose-metabolizing enzymes, consistent with previous reports showing that molecular chaperones boost heterologous pathway performance in yeast [57,58]. By contrast, overexpression of the cytosolic catalase *CTT1* and the mitochondrial peroxiredoxin *PRX1* improved H_2_O_2_ tolerance as expected [59,60], but *PRX1* overexpression unexpectedly reduced acetic acid tolerance (Figure S5), highlighting the intricate connection between mitochondrial redox homeostasis and weak acid resistance [61]. These results identify *SSB2* as a potential engineering target. Meanwhile, the challenge of enhancing stress tolerance through antioxidant pathways is underscored by the trade-off between oxidative and acetic acid resistance associated with *PRX1* overexpression. However, other prominent genetic variations identified in the YB-2625 genome, such as those in the *FLO* flocculation genes, *HXT* transporters, and *MIG* glucose repressors, were not included in the present functional validation. In addition to these metabolic and structural candidates, numerous transcription factors and epigenetic regulators exhibiting either non-synonymous mutations or significant differential expression also warrant future investigation. These loci represent promising candidates for further functional dissection to fully elucidate the complex regulatory network underpinning the robust phenotype of YB-2625.

**Figure 6:**
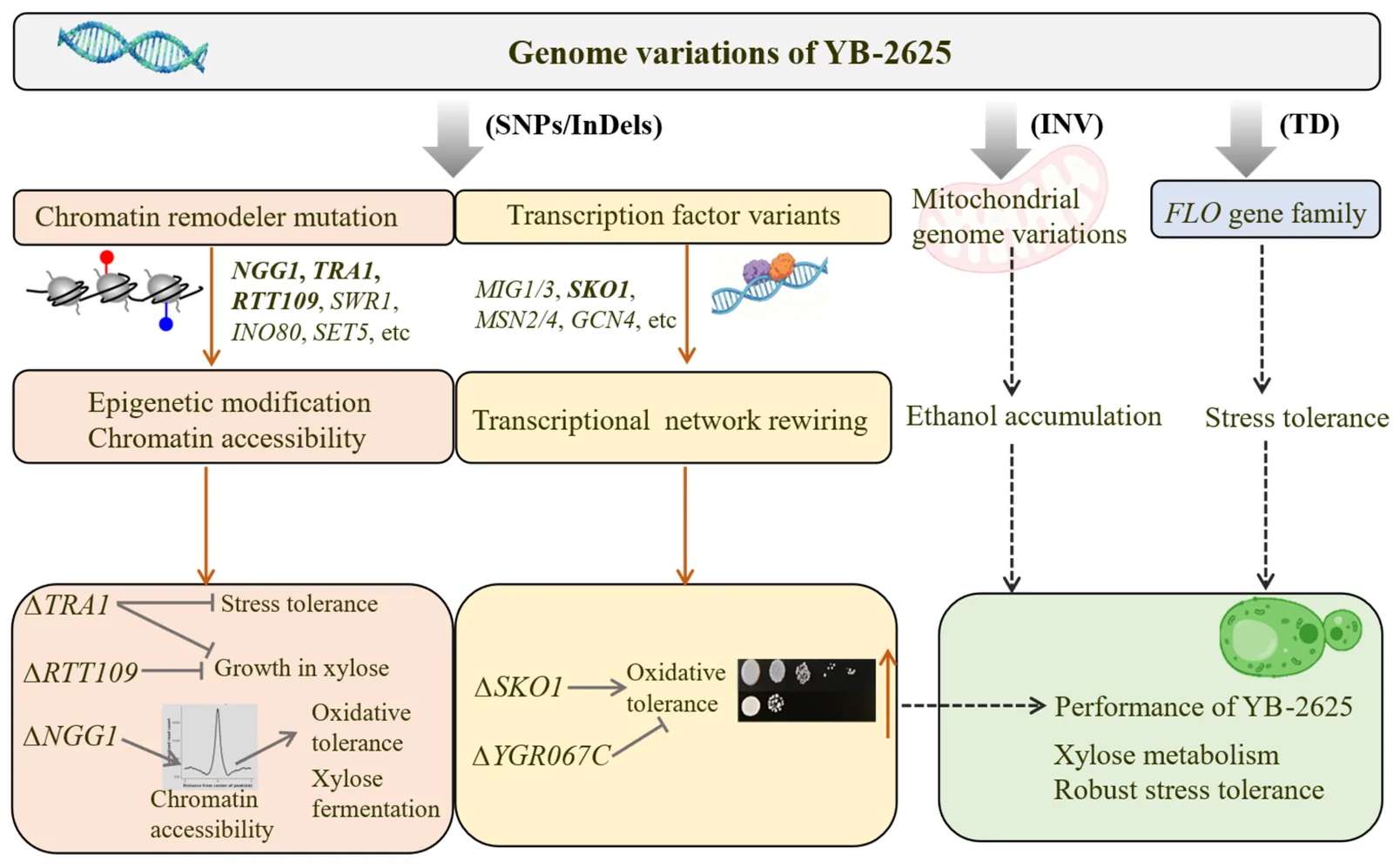
Multi-layered molecular mechanisms underlying the superior fermentation performance of yeast strain YB-2625. Genome-level variations, including SNPs/InDels, mitochondrial inversions (INV) and tandem duplications (TD), form the genetic foundation of YB-2625. Solid red arrows indicate partial genes with experimental validation, and dashed black arrows indicate genes inferred from multi-omics data. Functional validation through gene knockout assays confirmed the contributions of chromatin remodelers and stress regulators to xylose metabolism and oxidative defense. T-shaped arrows and solid gray arrows represent phenotypic inhibition and improvement caused by the genetic modifications. *TRA1* acted as a global regulator with growth-coupled effects. *RTT109* specifically modulated xylose metabolism, while *SKO1* contributed to oxidative stress defense. Deletion of *NGG1* enhances xylose utilization and contributes to oxidative stress defense.

In summary, the robust phenotype of YB-2625 does not arise from a single super mutation, but rather from coordinated rewiring of specific regulatory nodes across the nuclear and mitochondrial genomes (Figure 6). At the epigenetic level, the natural down-regulation of *NGG1*, combined with its strain-specific C-terminal missense mutations, reshapes chromatin accessibility at loci governing carbohydrate metabolism, stress defense, and amino acid biosynthesis. *TRA1* emerges as a central coactivator regulator, as its disruption simultaneously compromises both multi-stress tolerance and xylose assimilation, thereby mechanistically linking chromatin remodeling through SAGA and NuA4 to carbon metabolism. In addition to chromatin regulators, strain-specific elements further fine-tune the phenotype. The presence of *SSB2*, which is absent in S288C, appears to enhance xylose-to-ethanol conversion, potentially through cotranslational protein folding, while deletions of *SKO1* and *YGR067C* suggest the involvement of these genes in specialized oxidative stress defense pathways. At the metabolic level, variations in *HXT* transporters and *MIG1/MIG3* repressors alleviate glucose repression, and tandem duplications in the *FLO* gene family reinforce acetic acid tolerance through flocculation. Concomitantly, the extensive mitochondrial inversions affecting *COX2*, *COB*, and *OLI1* are proposed to redirect carbon flux from respiration toward fermentation, further promoting xylose utilization and reducing byproduct accumulation.

This integrated model encompasses allele-specific epigenetic modifiers such as Ngg1, essential coactivators such as Tra1, strain specific chaperones such as Ssb2, and organellar genome remodeling, thereby providing a multi-layered genetic foundation for the enhanced robustness of YB-2625. However, we acknowledge that the current conclusions are drawn primarily from genomic, transcriptomic, and chromatin accessibility data; functional validation at the protein level and structural resolution of the proposed regulatory interactions warrant further investigation. Nevertheless, this integrative omics approach offers a useful roadmap for dissecting complex polygenic traits in natural yeast isolates and guiding rational strain improvement.

## 4. Conclusions

The multi-omics analyses in this study reveal that the superior xylose fermentation and stress tolerance of YB-2625 arise from coordinated variations across multiple regulatory layers, including allele specific epigenetic modifiers, essential transcriptional coactivators, strain-specific chaperones, and organellar genome remodeling. Functional validation demonstrates that optimal xylose fermentation requires a delicate balance in the activity of chromatin regulators such as Rtt109 and Ngg1. These findings highlight natural yeast diversity as a valuable resource for discovering non-intuitive engineering targets and provide a rational framework for reprogramming endogenous regulatory networks in next-generation industrial strains for lignocellulosic bioconversion.

## Figures and Tables

**Table 1:** Representative genes with concordant changes in chromatin accessibility and transcription in *NGG1* deletion mutant relative to the control YRH396h.

Functional Category	Up-Regulated Genes	Down-Regulated Genes
Stress tolerance	—	*GRE1*, *DDR2*, *HSP12*, *CTT1*, *GPX1*, *GTT1*, *ALD3*, *ALD4*, *GLO1*, *CRS5*, *MHO1*, *TMC1*
Redox homeostasis	*OYE2*, *GRX5*, *TRX1*	—
Carbon metabolism	—	*TKL2*, *ICL1*, *ATH1*, *GLC3*, *CYB2*
Cell wall organization	*FLO5*, *HSP150*, *GAS1*, *SCW4*	*TIP1*, *ECM3*, *CHS1*, *PMP3*
Membrane transport and signaling	*PMA1*, *STT4*	*ENA1*, *ATO2*, *ATO3*, *HXT2*, *HXT5*
Lipid metabolism	*HFA1*	*PXA2*, *OSH2*
Energy metabolism	*ATP1*	*—*
Epigenetic and transcriptional regulation	—	*GIS1*, *NRG2*, *EPL1*

Dashes (—) indicate that no significant co-regulated genes were identified in that category.

## Data Availability

The genomic data are publicly available under NCBI BioProject accession number PRJNA1492256. The transcriptomic and ATAC-Seq datasets are deposited under BioProject PRJNA1514219 and PRJNA1497054, respectively. All processed data supporting the conclusions are included in the article and its Supplementary Materials.

## Supplementary Materials

The following supporting information can be downloaded at: https://www.mdpi.com/article/10.3390/jof12090631/s1, Figure S1: Thermotolerance of various yeast strains assessed by spot assay; Figure S2: Gene Ontology (GO) enrichment analysis of genes carrying InDels; Figure S3: Mitochondrial genome structural variations and respiratory activity assay of YB-2625 compared to control strain S288C; Figure S4: Serial dilution spot growth assay for stress tolerance of wild-type YRH396h and Δ*RTT109* mutant; Figure S5: Evaluation of stress tolerance and fermentation performance of *S. cerevisiae* strains; Table S1: Yeast strains used in this study; Table S2: Primers used for PCR validation of selected genes; Table S3: Primers for gene deletion and overexpression; Table S4: Candidate genes screened by combined genomic variant analysis and transcriptome profiling with genetic manipulation validation.

## References

[1] Chen Z., Chen L., Khoo K.S., Gupta V.K., Sharma M., Show P.L., Yap P.S. (2023). Exploitation of lignocellulosic-based biomass biorefinery: A critical review of renewable bioresource, sustainability and economic views. Biotechnol. Adv..

[2] Bukhari I., Haq F., Kiran M., Aziz T., Mehmood S., Haroon M. (2025). Lignocellulosic biomass as a renewable resource: Driving second-generation biofuel innovation from agricultural waste. Biomass Bioenergy.

[3] Vargas B.O., Dos Santos J.R., Pereira G.A.G., de Mello F.D.S.B. (2023). An atlas of rational genetic engineering strategies for improved xylose metabolism in *Saccharomyces cerevisiae*. PeerJ.

[4] Sun D., Wu L., Lu X., Li C., Xu L., Li H., He D., Yu A., Yu T., Zhao J. (2025). Engineering transcriptional regulatory networks for improving second-generation fuel ethanol production in *S. cerevisiae*. Synth. Syst. Biotechnol..

[5] Sharma N.K., Behera S., Arora R., Kumar S., Sani R.K. (2018). Xylose transport in yeast for lignocellulosic ethanol production: Current status. J. Biosci. Bioeng..

[6] Taveira I.C., Fier Í., Nogueira K.M.V., Maues D.B., Santos L.V., Silva R.N. (2026). Docking-guided and phosphomimetic rational engineering of glucose-insensitive xylose transporters from *Trichoderma reesei*. Microb. Cell Fact..

[7] Dos Santos L.V., Neitzel T., Lima C.S., de Carvalho L.M., de Lima T.B., Ienczak J.L., Corrêa T.L.R., Pereira G.A.G. (2025). Engineering cellular redox homeostasis to optimize ethanol production in xylose-fermenting *S. cerevisiae*. Microbiol. Res..

[8] Sahoo A., Das P.K., Veeranki V.D., Patra S. (2025). Engineered *Saccharomyces cerevisiae* for sustainable biobased fuel production: Overcoming bottlenecks and implementing strategies. Renew. Sustain. Energy Rev..

[9] Wagner E.R., Gasch A.P. (2023). Advances in *S. cerevisiae* engineering for xylose fermentation and biofuel production: Balancing growth, metabolism, and defense. J. Fungi.

[10] Patiño M.A., Ortiz J.P., Velásquez M., Stambuk B.U. (2019). d-Xylose consumption by nonrecombinant *Saccharomyces cerevisiae*: A review. Yeast.

[11] Filleton F., Chuffart F., Nagarajan M., Bottin-Duplus H., Yvert G. (2015). The complex pattern of epigenomic variation between natural yeast strains at single-nucleosome resolution. Epigenet. Chromatin.

[12] Bleykasten-Grosshans C., Fabrizio R., Friedrich A., Schacherer J. (2021). Species-wide transposable element repertoires retrace the evolutionary history of the *Saccharomyces cerevisiae* host. Mol. Biol. Evol..

[13] Loegler V., Thiele P., Teyssonnière E., Tsouris A., Brach G., Cruaud C., Payen E., Engelen S., Dunham M.J., Hou J. (2025). From genotype to phenotype with 1,086 near telomere-to-telomere yeast genomes. Nature.

[14] Papapetridis I., Verhoeven M.D., Wiersma S.J., Goudriaan M., van Maris A.J.A., Pronk J.T. (2018). Laboratory evolution for forced glucose-xylose co-consumption enables identification of mutations that improve mixed-sugar fermentation by xylose-fermenting *Saccharomyces cerevisiae*. FEMS Yeast Res..

[15] Long Y., Han X., Meng X., Xu P., Tao F. (2024). A robust yeast chassis: Comprehensive characterization of a fast-growing *Saccharomyces cerevisiae*. mBio.

[16] Wang H., Nielsen J., Zhou Y.J., Lu H. (2025). Yeast adapts to diverse ecological niches driven by genomics and metabolic reprogramming. Proc. Natl. Acad. Sci. USA.

[17] Cheng C., Wang W.B., Sun M.L., Tang R.Q., Bai L., Alper H.S., Zhao X.Q. (2022). Deletion of *NGG1* in a recombinant *Saccharomyces cerevisiae* improved xylose utilization and affected transcription of genes related to amino acid metabolism. Front. Microbiol..

[18] Yuan B., Wang W.B., Wang X.Q., Liu C.G., Hasunuma T., Kondo A., Zhao X.Q. (2024). The chromatin remodeler Ino80 regulates yeast stress tolerance and cell metabolism through modulating nitrogen catabolite repression. Int. J. Biol. Macromol..

[19] Xiao X.Y., Li B., Xia Z.Y., Zhang Q., Xie C.Y., Tang Y.Q. (2025). Regulatory mechanism of Haa1p and Hap4p in *Saccharomyces cerevisiae* to mixed acetic acid and formic acid when fermenting mixed glucose and xylose. Microb. Cell Fact..

[20] Watcharawipas A., Runguphan W., Khamwachirapithak P., Laothanachareon T. (2025). Integrating yeast biodiversity and machine learning for predictive metabolic engineering. FEMS Yeast Res..

[21] Cheng C., Tang R.Q., Xiong L., Hector R.E., Bai F.W., Zhao X.Q. (2018). Association of improved oxidative stress tolerance and alleviation of glucose repression with superior xylose-utilization capability by a natural isolate of *Saccharomyces cerevisiae*. Biotechnol. Biofuels.

[22] Hector R.E., Mertens J.A., Nichols N.N. (2023). Metabolic engineering of a stable haploid strain derived from lignocellulosic inhibitor tolerant *Saccharomyces cerevisiae* natural isolate YB-2625. Biotechnol. Biofuels Bioprod..

[23] Hector R.E., Dien B.S., Cotta M.A., Qureshi N. (2011). Engineering industrial *Saccharomyces cerevisiae* strains for xylose fermentation and comparison for switchgrass conversion. J. Ind. Microbiol. Biotechnol..

[24] Gietz R.D., Schiestl R.H. (2007). High-efficiency yeast transformation using the LiAc/SS carrier DNA/PEG method. Nat. Protoc..

[25] Schep A.N., Buenrostro J.D., Denny S.K., Schwartz K., Sherlock G., Greenleaf W.J. (2015). Structured nucleosome fingerprints enable high-resolution mapping of chromatin architecture within regulatory regions. Genome Res..

[26] Wang L., Zhao X.Q., Xue C., Bai F.W. (2013). Impact of osmotic stress and ethanol inhibition in yeast cells on process oscillation associated with continuous very-high-gravity ethanol fermentation. Biotechnol. Biofuels.

[27] Guaragnella N., Bettiga M. (2021). Acetic acid stress in budding yeast: From molecular mechanisms to applications. Yeast.

[28] Vasylyshyn R., Ruchala J., Dmytruk K., Sibirny A. (2026). Thermotolerant yeasts and their biotechnological applications. Trends Biotechnol..

[29] Van Mulders S.E., Christianen E., Saerens S.M., Daenen L., Verbelen P.J., Willaert R., Verstrepen K.J., Delvaux F.R. (2009). Phenotypic diversity of Flo protein family-mediated adhesion in *Saccharomyces cerevisiae*. FEMS Yeast Res..

[30] Zhang X., Dai Y., Zhao X.Q., Liu C.G., Wang Z., Bai F.W. (2026). Omics analyses decoding mechanisms underlying the self-flocculating phenotype of yeast cells and stress tolerance for robust production. Metab. Eng..

[31] Zhang Y., Xiao D.G., Zhang C.Y., Sun X., Wu M.Y. (2011). Effect of *MIG1* gene deletion on glucose repression in baker’s yeast. Adv. Mater. Res..

[32] Peng B., Shen Y., Li X., Chen X., Hou J., Bao X. (2012). Improvement of xylose fermentation in respiratory-deficient xylose-fermenting *Saccharomyces cerevisiae*. Metab. Eng..

[33] Wang W.B., Tang R.Q., Yuan B., Wang Y., Liu G.D., Li D.M., Zhang H.J., Zhao X.Q., Bai F.W. (2025). Engineering chromatin regulation of xylose utilization in budding yeast *Saccharomyces cerevisiae* for efficient bioconversion. ACS Synth. Biol..

[34] Zhang M.M., Yuan B., Wang Y.T., Zhang F.L., Liu C.G., Zhao X.Q. (2024). Differential protein expression in Set5p-mediated acetic acid stress response and novel targets for engineering yeast stress tolerance. J. Proteome Res..

[35] Orzechowski Westholm J., Tronnersjö S., Nordberg N., Olsson I., Komorowski J., Ronne H. (2012). Gis1 and Rph1 regulate glycerol and acetate metabolism in glucose depleted yeast cells. PLoS ONE.

[36] Srinivasan R., Walvekar A.S., Rashida Z., Seshasayee A., Laxman S. (2020). Genome-scale reconstruction of Gcn4/ATF4 networks driving a growth program. PLoS Genet..

[37] Natarajan K., Meyer M.R., Jackson B.M., Slade D., Roberts C., Hinnebusch A.G., Marton M.J. (2001). Transcriptional profiling shows that Gcn4p is a master regulator of gene expression during amino acid starvation in yeast. Mol. Cell. Biol..

[38] von Plehwe U., Berndt U., Conz C., Chiabudini M., Fitzke E., Sickmann A., Petersen A., Pfeifer D., Rospert S. (2009). The Hsp70 homolog Ssb is essential for glucose sensing via the *SNF1* kinase network. Genes Dev..

[39] Brown C.E., Howe L., Sousa K., Alley S.C., Carrozza M.J., Tan S., Workman J.L. (2001). Recruitment of HAT complexes by direct activator interactions with the ATM-related Tra1 subunit. Science.

[40] Knutson B.A., Hahn S. (2011). Domains of Tra1 important for activator recruitment and transcription coactivator functions of SAGA and NuA4 complexes. Mol. Cell. Biol..

[41] Elías-Villalobos A., Fort P., Helmlinger D. (2019). New insights into the evolutionary conservation of the sole PIKK pseudokinase Tra1/TRRAP. Biochem. Soc. Trans..

[42] Lin Y.Y., Lu J.Y., Zhang J., Walter W., Dang W., Wan J., Tao S.C., Qian J., Zhao Y., Boeke J.D. (2009). Protein acetylation microarray reveals that NuA4 controls key metabolic target regulating gluconeogenesis. Cell.

[43] Hoke S.M., Guzzo J., Andrews B., Brandl C.J. (2008). Systematic genetic array analysis links the *Saccharomyces cerevisiae* SAGA/SLIK and NuA4 component Tra1 to multiple cellular processes. BMC Genet..

[44] Mormino M., Lenitz I., Siewers V., Nygård Y. (2022). Identification of acetic acid sensitive strains through biosensor-based screening of a *Saccharomyces cerevisiae* CRISPRi library. Microb. Cell Fact..

[45] Driscoll R., Hudson A., Jackson S.P. (2007). Yeast Rtt109 promotes genome stability by acetylating histone H3 on lysine 56. Science.

[46] Cheng C., Zhao X., Zhang M., Bai F. (2016). Absence of Rtt109p, a fungal-specific histone acetyltransferase, results in improved acetic acid tolerance of *Saccharomyces cerevisiae*. FEMS Yeast Res..

[47] Kobayashi Y., Inai T., Mizunuma M., Okada I., Shitamukai A., Hirata D., Miyakawa T. (2008). Identification of Tup1 and Cyc8 mutations defective in the responses to osmotic stress. Biochem. Biophys. Res. Commun..

[48] Heredia M.Y., Ikeh M.A.C., Gunasekaran D., Conrad K.A., Filimonava S., Marotta D.H., Nobile C.J., Rauceo J.M. (2020). An expanded cell wall damage signaling network is comprised of the transcription factors Rlm1 and Sko1 in *Candida albicans*. PLoS Genet..

[49] Bjurström E.Y., Gower A.H., Lasin P., Savolainen O.I., Tiukova I.A., King R.D. (2026). Investigating uncharacterised genes in *Saccharomyces cerevisiae* using robot scientists. Sci. Rep..

[50] Stavropoulos P., Nagy V., Blobel G., Hoelz A. (2008). Molecular basis for the autoregulation of the protein acetyl transferase Rtt109. Proc. Natl. Acad. Sci. USA.

[51] Horiuchi J., Silverman N., Marcus G.A., Guarente L. (1995). *ADA3*, a putative transcriptional adaptor, consists of two separable domains and interacts with *ADA2* and *GCN5* in a trimeric complex. Mol. Cell. Biol..

[52] Saleh A., Lang V., Cook R., Brandl C.J. (1997). Identification of native complexes containing the yeast coactivator/repressor proteins *NGG1/ADA3* and *ADA2*. J. Biol. Chem..

[53] Brandl C.J., Martens J.A., Margaliot A., Stenning D., Furlanetto A.M., Saleh A., Hamilton K.S., Genereaux J. (1996). Structure/functional properties of the yeast dual regulator protein *NGG1* that are required for glucose repression. J. Biol. Chem..

[54] Huang J., Dai W., Xiao D., Xiong Q., Liu C., Hu J., Ge F., Yu X., Li S. (2022). Acetylation-dependent SAGA complex dimerization promotes nucleosome acetylation and gene transcription. Nat. Struct. Mol. Biol..

[55] Tan Y.S., Wang L., Wang Y.Y., He Q.E., Liu Z.H., Zhu Z., Song K., Li B.Z., Yuan Y.J. (2021). Protein acetylation regulates xylose metabolism during adaptation of Saccharomyces cerevisiae. Biotechnol. Biofuels.

[56] Willmund F., del Alamo M., Pechmann S., Chen T., Albanèse V., Dammer E.B., Peng J., Frydman J. (2013). The cotranslational function of ribosome-associated Hsp70 in eukaryotic protein homeostasis. Cell.

[57] Hou J., Jiao C., Peng B., Shen Y., Bao X. (2016). Mutation of a regulator Ask10p improves xylose isomerase activity through up-regulation of molecular chaperones in *Saccharomyces cerevisiae*. Metab. Eng..

[58] Temer B., Dos Santos L.V., Negri V.A., Galhardo J.P., Magalhães P.H.M., José J., Marschalk C., Corrêa T.L.R., Carazzolle M.F., Pereira G.A.G. (2017). Conversion of an inactive xylose isomerase into a functional enzyme by co-expression of GroEL-GroES chaperonins in *Saccharomyces cerevisiae*. BMC Biotechnol..

[59] Abbott D.A., Suir E., Duong G.H., de Hulster E., Pronk J.T., van Maris A.J. (2009). Catalase overexpression reduces lactic acid-induced oxidative stress in *Saccharomyces cerevisiae*. Appl. Environ. Microbiol..

[60] Pedrajas J.R., Miranda-Vizuete A., Javanmardy N., Gustafsson J.A., Spyrou G. (2000). Mitochondria of *Saccharomyces cerevisiae* contain one-conserved cysteine type peroxiredoxin with thioredoxin peroxidase activity. J. Biol. Chem..

[61] Dong Y., Hu J., Fan L., Chen Q. (2017). RNA-Seq-based transcriptomic and metabolomic analysis reveal stress responses and programmed cell death induced by acetic acid in *Saccharomyces cerevisiae*. Sci. Rep..

