# Supplementary material for: Multi-Omics Dissection and Functional Validation of Candidate Regulators Modulating Stress Tolerance and Xylose Utilization in the Natural Yeast Strain YB-2625
Source: J Fungi (Basel). 2026 Aug 23;12(9):631. doi: 10.3390/jof12090631 (PMC13608086; doi:10.3390/jof12090631)
Supplement: Supplementary file 1 [file jof-12-00631-s001.zip › jof-4461309-supplementary.pdf]

Supplementary data for

Multi-Omics Dissection and Functional Validation of Candidate Regulators Modulating Stress Tolerance and Xylose Utilization in the Natural Yeast Strain YB-2625

Cheng Cheng [1], Teng-Fei Wu [2], Hong-Lei Mao [1], Wei-Bin Wang [2] and Xin-Qing Zhao [2,*]

[1] School of Biotechnology and Food Engineering, Hefei Normal University, Hefei 230601, China

[2] State Key Laboratory of Microbial Metabolism, School of Life Sciences and Biotechnology, Shanghai Jiao Tong University, Shanghai 200240, China

* Correspondence: xqzhao@sjtu.edu.cn; Tel.: +86-21-34206673

Table S1 Yeast strains used in this study

Yeast strain	Genotype	Source
YB-2625	*S. cerevisiae* isolated from bagasse	NRRL
Y-2034	*S. cerevisiae* isolated from wine	NRRL
Y-1528	*S. cerevisiae*, natural galactose-assimilating isolate	NRRL
S288c	*S. cerevisiae*, laboratory strain	Stored in our lab
*Sc*6525	*S. cerevisiae*, wild-type, diploid, industrial strain	Provided by Dr. Jana Otrubo
*Sc*4126	*S. cerevisiae*, wild-type, diploid, industrial strain	Provided by Dr. Jana Otrubo
CEN.PK113-5D	*S. cerevisiae* (*MATa; ura3-52; HIS3; LEU2; TRP1; MAL2-8^c SUC2*)	Eurocarf
CEN.PK113-7D	*S. cerevisiae* (*MATa; URA3; HIS3; LEU2; TRP1; MAL2-8^c SUC2*)	Eurocarf
KAM-2	*S. cerevisiae* (*MATa; ura3*)	Stored in our lab
L2612	*S. cerevisiae* (*Mata; leu2, trp1, ura3*)	Provided by Dr. Jin-Ho Seo
BY4741	*S. cerevisiae* (*MATa*, *his3⊿1*, *leu2⊿0*, *met15⊿0* and *ura3⊿0*)	Eurocarf
SPSC01	Self-flocculating yeast, wild-type, diploid, industrial strain	Stored in our lab
YRH396h	*S. cerevisiae*, haploid derivative of the diploid xylose-fermenting recombinant strain YRH396	Stored in our lab

Table S2 Primers used for PCR validation of selected genes

Primer name	Sequence (5′→3′)	Length of PCR product (bp)
ZWF1-F	ATGAGTGAAGGCCCCGTCAAA	444
ZWF1-R	ATTCTCTGCGTACACACGAC	
FPS1-F	CTGTCCAGTGAATCTGTTCA	508
FPS1-R	ACCTACTTCTCCTTCTCGAT	
ZAP1-F	ATGGATGCGTTGACTCCCA	792
ZAP1-R	GTGAGAGTCGTGATTGGTAC	
MSN4-F	CGAGCCAAACGGATTGATGG	1692
MSN4-R	TTCAAGTGCTCACTGCGTCT	
ADR1-F	GACGCAGACTTATTGTCGTC	882
ADR1-R	CAATTCACTCACAGCTGGCA	

Table S3 Primers for gene deletion and overexpression

**Primer name**	**Sequence 5′-3′**
Δ*TRA1*-F	ATGTCACTCACTGAGCAGATCGAGCAATTCGCCAGTAGGTCAGCGACATGGAGGCCCAG
Δ*TRA1*-R	CCGTTACCAATAAGTATACGTTCGTCAAACAGATAGTCCACGTCCCAAAACCTTCTCAA
Δ*YGR067C* -F	ATGGCTGCGGGTCAAAAAAAATATATATGTTCCTTTTGTTCAGCGACATGGAGGCCCAG
Δ*YGR067C*-R	ACTGGAGGATGCCACGGTGAATGGCTTCAGAATATCTTGACGTCCCAAAACCTTCTCAA
Δ*SKO1* -F	ATGTCAAGCGAGGAACGCTCGAGACAACCAAGTACTGTTTCAGCGACATGGAGGCCCAG
Δ*SKO1* -R	AGGGTATTATGCCGCATAGCTTCCCAATGACCTGAGTTAGCGTCCCAAAACCTTCTCAA
Δ*RTT109*-F	ATGTCACTGAATGACTTCCTAAGTTCCGTGCTACCTGTCACAGCGACATGGAGGCCCAG
Δ*RTT109*-R	TAGCATTGTTATCGCCAACGTGTTGATGTTGCTTGCAGGACGTCCCAAAACCTTCTCAA
hph-in-R	TGGTCAAGACCAATGCGGAG
NGG1(GOE)-F	GCGTCGACATGCCTAGACATGGAAGAAG
NGG1(GOE)-R	CCTTAATTAATTAATTTAGTTCCACGTCCTTATTAGTG
RTT109(GOE)-F	GCGTCGACATGTCACTGAATGACTTCCT
RTT109(GOE)-R	CCTTAATTAATCAAGTTTTAGGCAAGGCTTTAGC
H-*PRX1*-F	CCAAGCTTATGTTTAGTAGAATTTGTAGC
P-*PRX1*-R	CCTTAATTAATTATTTCGACTTGGTGAATC
H-*CTT1*-F	CCAAGCTTATGAACGTGTTCGGTAAAAAAG
P-*CTT1*-R	CCTTAATTAATTAATTGGCACTTGCAATGG
S-*SSB2*-F	GCGTCGACATGGCTGAAGGTGTTTTCCAAG
P-*SSB2*-R	CCTTAATTAATTAACGAGAAGACATGGCCTTG

Table S4 Candidate genes screened by combined genomic variant analysis and transcriptome profiling with genetic manipulation validation

**Gene**	**Mutation Type**	log2 Ratio **(**YB-XG vs SC-XG)	P-value	log2 Ratio **(**YB-X vs SC-X)	P-value	log2 Ratio **(**SC-X vs SC-XG)	P-value	log2 Ratio **(**YB-X vs YB-XG)	P-value	**Function**
NGG1	SNPs	-	-	-0.61	8.33E-08	-	-	-0.49	1.60E-07	Subunit of chromatin modifying histone acetyltransferase complexes; member of the SAGA complex
TRA1	SNPs	-0.43	7.44E-09	1.41	2.38E-24	-1.56	2.01E-65	0.34	0.00659	Subunit of SAGA and NuA4 histone acetyltransferase complexes
YGR067C	Indels-frame shifted	-0.51	8.06E-07	-1.45	1.74E-10	-	-	-0.70	0.002433	Putative protein of unknown function; contains a zinc finger motif similar to that of Adr1p
SKO1	Indels-insertion	-0.30	2.95E-06	0.20	0.08093	-1.18	1.08E-35	-0.64	1.78E-16	Basic leucine zipper transcription factor, cytosolic and nuclear protein involved in osmotic and oxidative stress responses
RTT109	SNPs	-0.50	0.001264	-1.62	3.31E-13	-0.74	9.24E-07	-1.81	1.47E-15	Histone acetyltransferase
PRX1	SNPs	-	-	0.43	0.0004726	-	-	0.36	1.46E-06	Mitochondrial peroxiredoxin with thioredoxin peroxidase activity
CTT1	SNPs	-2.47	7.56E-77	-5.51	1.00E-100	-1.89	0	-2.70	1.36E-53	Cytosolic catalase T, has a role in protection from oxidative damage by hydrogen peroxide
SSB2	SNPs	1.00	3.77E-33	-0.33	0.01726	0.74	3.93E-11	-0.54	6.96E-06	Cytoplasmic ATPase that is a ribosome-associated molecular chaperon

Note: $\log_2$ Ratio(YB-XG vs SC-XG): Gene expression fold change of YB-2625 relative to S288c under mixed sugar fermentation stage; **$\log_2$ Ratio(YB-X vs SC-X)**: Transcriptional difference between YB-2625 and S288c in xylose fermentation stage; **$\log_2$ Ratio(SC-X vs SC-XG)**: Expression variation of S288c cultured in xylose compared to mixed sugar; **$\log_2$ Ratio(YB-X vs YB-XG)**: Expression changes of YB-2625 in xylose versus mixed sugar fermentation. Mutation types cover SNPs and Indels (frameshift and insertion mutations).

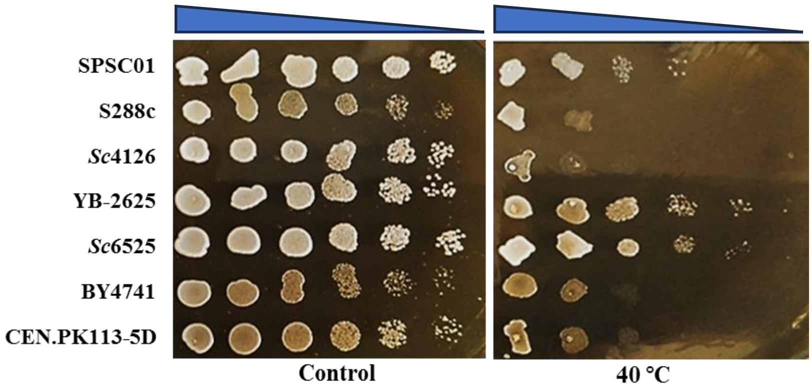

Figure S1. Thermotolerance of various yeast strains assessed by spot assay. Yeast cell growth ability was evaluated on YPD agar medium by ten-fold dilution at 40°C, and YPD medium incubated at 30°C was used as the control. The experiments were performed in triplicate with reproducible results.

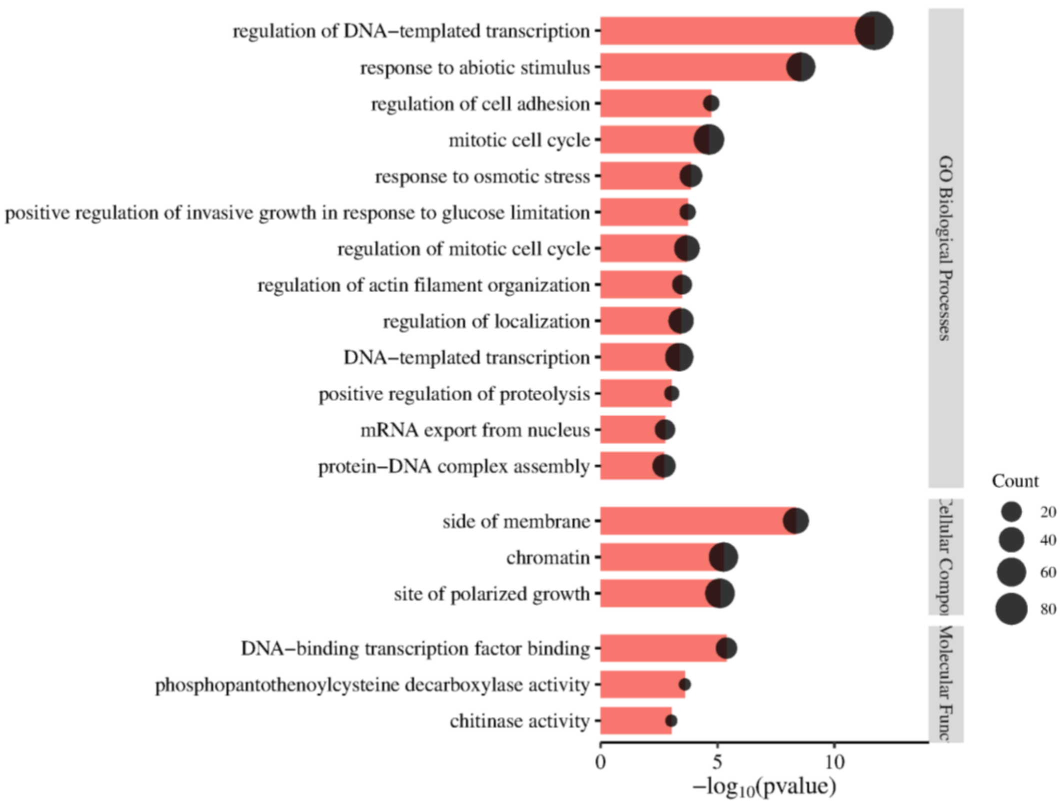

Figure S2. Gene Ontology (GO) enrichment analysis of genes carrying InDels. The top significantly enriched GO terms are displayed, ranked by $-\log_{10}$ value. Dot size represents the number of genes annotated to each term.

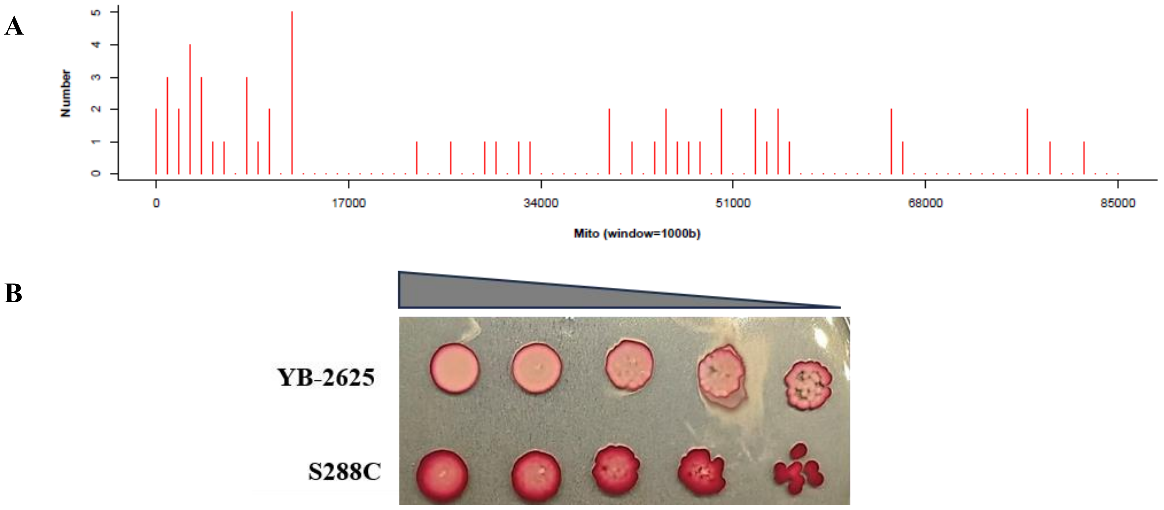

Figure S3. Mitochondrial genome structural variations and respiratory activity assay of YB-2625 compared to control strain S288C. A. Distribution of inversion (INV) sites in the mitochondrial genome of YB-2625 relative to S288C. The genome was scanned in 1-kb windows. The x-axis indicates position (kb); the y-axis indicates the number of INV sites per window. B. TTC (2,3,5-triphenyltetrazolium chloride) spot-dilution assay for evaluating cellular respiratory intensity. A series of gradient dilutions by 100, 500, 1000, 5000, and 10000-fold of *S. cerevisiae* S288C and YB-2625 were spotted onto YPD agar plates supplemented with 5 g/L xylose and cultured at 30 °C for 48 h. Plates were then overlaid with 0.1% (w/v) TTC solution and incubated at 30 °C in the dark for 2 h. All experiments were performed in three biological replicates.

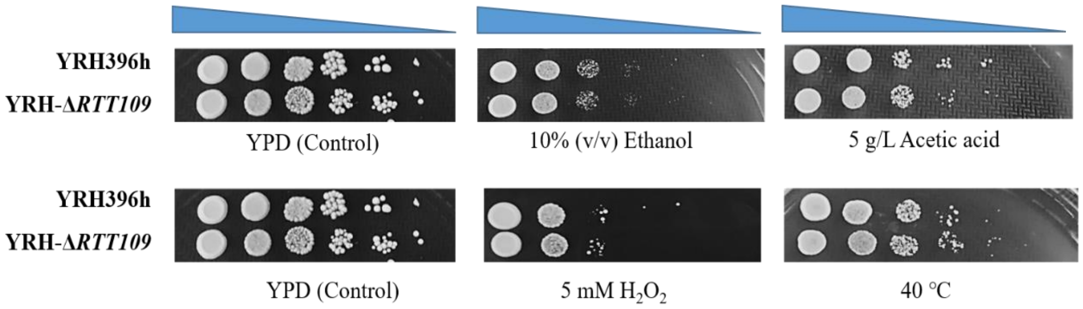

Figure S4. Serial dilution spot growth assay for stress tolerance of wild-type YRH396h and Δ*RTT109* mutant. Yeast cells were serially diluted and spotted on control YPD plates or YPD plates with 10% ethanol, 5 g/L acetic acid, 5 mM H_2O_2, or cultured at 40 °C. Gradient dilution is indicated by the blue triangle above each panel.

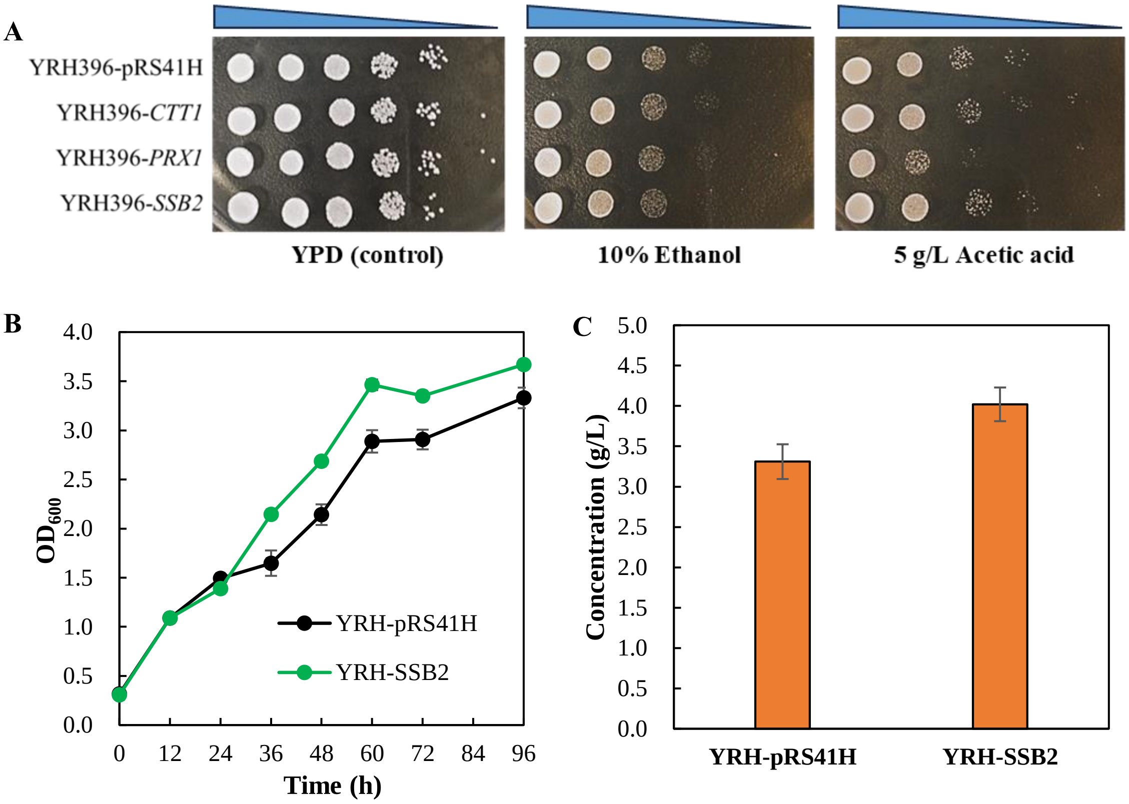

Figure S5. Evaluation of stress tolerance and fermentation performance of *S. cerevisiae* strains. (A) Spot assays assessing the growth of strains YRH396-pRS41H (control), YRH396-*CTT1*, YRH396-*PRX1*, and YRH396-*SSB2* under different stress conditions. Serial dilutions of cell cultures were spotted onto YPD plates (control), YPD supplemented with 10% ethanol and 5 g/L acetic acid, respectively. (B) and (C) Growth curves (OD_{600}) of YRH396-pRS41H and YRH396-*SSB2* during 96 h fermentation in 40 g/L xylose medium and final ethanol production of the two strains at the end of fermentation. No significant difference was observed ($P > 0.05$).
